# Changes and classification in myocardial contractile function in the left ventricle following acute myocardial infarction

**DOI:** 10.1098/rsif.2017.0203

**Published:** 2017-07-26

**Authors:** Hao Gao, Andrej Aderhold, Kenneth Mangion, Xiaoyu Luo, Dirk Husmeier, Colin Berry

**Affiliations:** 1 School of Mathematics and Statistics, University of Glasgow, Glasgow, UK; 2 British Heart Foundation Glasgow Cardiovascular Research Centre, University of Glasgow, Glasgow, UK

**Keywords:** myocardial infarction, contractility, cardiac modelling, machine learning, statistical inference, immersed boundary method

## Abstract

In this research, we hypothesized that novel biomechanical parameters are discriminative in patients following acute ST-segment elevation myocardial infarction (STEMI). To identify these biomechanical biomarkers and bring computational biomechanics ‘closer to the clinic’, we applied state-of-the-art multiphysics cardiac modelling combined with advanced machine learning and multivariate statistical inference to a clinical database of myocardial infarction. We obtained data from 11 STEMI patients (ClinicalTrials.gov NCT01717573) and 27 healthy volunteers, and developed personalized mathematical models for the left ventricle (LV) using an immersed boundary method. Subject-specific constitutive parameters were achieved by matching to clinical measurements. We have shown, for the first time, that compared with healthy controls, patients with STEMI exhibited increased LV wall active tension when normalized by systolic blood pressure, which suggests an increased demand on the contractile reserve of remote functional myocardium. The statistical analysis reveals that the required patient-specific contractility, normalized active tension and the systolic myofilament kinematics have the strongest explanatory power for identifying the myocardial function changes post-MI. We further observed a strong correlation between two biomarkers and the changes in LV ejection fraction at six months from baseline (the required contractility (*r* = − 0.79, *p* < 0.01) and the systolic myofilament kinematics (*r* = 0.70, *p* = 0.02)). The clinical and prognostic significance of these biomechanical parameters merits further scrutinization.

## 1. Background

Acute myocardial infarction (MI) is a common cause of premature morbidity and mortality. Although early survival post-ST segment elevation MI (STEMI) is improving [1], the longer term risk of heart failure remains persistently high [2]. The standard of care for assessing the initial severity of heart injury is left ventricular (LV) systolic function, and in particular, LV ejection fraction (LVEF) [1]. Nonetheless, global measures of LV pump function are simplistic [3,4], as compared to biomechanical parameters of pump function, such as myocardial contractility and stiffness.

There is a growing recognition that a computational approach for ventricular biomechanics, when integrated with clinical imaging, can provide insights into heart function and dysfunction [5,6]. For example, carefully designed models have been used to inform various improvement therapies post-MI [7].

Cardiac dynamics are complex multi-physics problems that involve dynamic blood flow, electrophysiology, nonlinear deformation and interactions among them [8]. Substantial effort has been devoted to developing computational models of the heart from simplified representations to more realistic image-derived cardiac models [8–12]. Among the computational frameworks that have been developed, the hybrid finite difference–finite-element version of the immersed boundary method (IB/FE) [13], one of the recent extensions of the immersed boundary (IB) method, has also been used by the authors' group to simulate LV dynamics with a hyperelastic representation of the fibre-reinforced myocardium [12,14,15].

One of the challenges for modelling the heart is that the myocardial constitutive parameters need to be determined prior to the modelling. Various approaches have been developed to estimate those parameters by matching the available clinical measurements (displacement, strain or pressure–volume curve) [16–20]. For example, Genet *et al.* [21] developed an image-based LV model by matching measured strain and volume data to construct a reference stress map which may be used in restoring LV stresses back to a normal level. Predicting the systolic stress inside LV wall will further require the incorporation of myocardial active contraction [8,9,22,23].

However, the biomechanics leading to LV adverse remodelling and heart failure remain incompletely understood [24], and controversy exists [25]. Modelling of diseased hearts has attracted a wide interest [12,26] in recent years. Using a Fung-type constitutive law [23], Guccione and colleagues found that the myofibre stress is increased in the infarct zone, and the contractility in the border zone is reduced. They suggested that the changed mechanical environment may lead to adverse remodelling post-MI [7,16,26,27]. By developing a biomechanical porcine heart model, Chabiniok *et al.* [28] estimated myocardial contractility from *in vivo* data at three time points after acute-MI, and found that the contractility in the remote regions of MI increased 10 days after acute-MI, followed by a further increase 38 days later. By simulating LV dynamics using a patient-specific clinical data, Gao *et al.* [12] reported that required myocardial contractility after acute-MI was much higher compared with a control heart, suggesting an increased use of the contractile reserve in the myocardial remote zone for the patient. Asner *et al.* [29] estimated peak contractility in a healthy volunteer and two patients with dilated cardiomyopathy using personalized mechanical LV models and, again paradoxically, the higher peak contractility was observed in the patients. However, myocardial contractility varied considerably for healthy and diseased hearts when estimated using computational models. The reasons for this variability are unclear but may relate to inter-individual variations, sample size or technical factors.

Biomechanical parameters, such as myocardial contractility and mechanical properties, are more directly linked with pump performance than global measures of systolic function (i.e. LVEF), and should have greater discriminative value for heart function and prognosis. However, direct measurements of these indices are very challenging, if not impossible *in vivo*, which limits the use of higher fidelity measures of pump function in clinic. Furthermore, it is not immediately obvious which of the biomechanical parameters have more clinical relevance or prognostic value [30]. The lack of a case-controlled study of the myocardial mechanical modelling between the healthy and diseased LVs makes it hard to identity the links between biomechanical characteristics and MI pathologies. The aim of this work is to study how myocardial contractility and pump function varies between healthy subjects and patients with a recent MI. Our questions are:

(1) Does myocardial mechanically function differ in patients versus controls?
(2) If so, what are the differences in the proposed biomechanical parameters?
(3) Can these parameters be used to classify myocardial contractile function and if so, which classification method is better?
(4) What are the clinical implications of these parameters?

To address these questions, we firstly carried out an extreme case–control study of LV biomechanical behaviours using the IB/FE method in a patient group with acute STEMI and a control group without history of cardiovascular disease. Secondly, we applied various machine learning and statistical classification methods to identify the potential biomarkers which may reflect myocardial function difference, and then evaluated their performance. Finally, we analysed potential associations between the proposed biomechanical biomarkers and the LV function recovery and remodelling at six months within the patient group.

## 2. Material and methods

### 2.1. Study design

The study involves an ‘extreme case–control’ approach [31] in order to enhance the statistical power of the analysis while using a limited sample size. The specific clinical focus of this work will be patients who have suffered a large, acute STEMI that is associated with reperfusion injury (i.e. no-reflow), which is a major, life-threatening cause of acute LV pump failure post-MI [32,33]. No-reflow is defined as an acute reduction in myocardial blood flow despite a patent epicardial coronary artery, and is independently associated with adverse remodelling and adverse outcome [34]. Twenty-seven healthy subjects and 11 patients with acute-MI were enrolled in this study. Cardiac magnetic resonance (CMR) scans were used to computationally infer biomechanical parameters that would otherwise not be available from *in vivo* measurements.

### 2.2. Mechanical left ventricle model

#### 2.2.1. Imaging-derived left ventricle model

**Figure 1.**
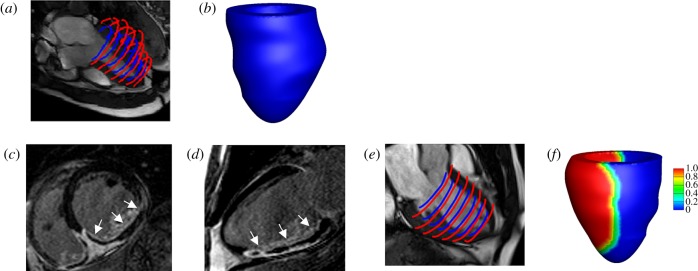
LV model constructions for one healthy control and one MI patient based on *in vivo* magnetic resonance imaging data. The healthy LV model: (*a*) LV wall segmentation; (*b*) reconstructed LV geometry. The MI model (*c*) short-axis LGE imaging, the infarct region is enhanced with micro-vascular obstruction appearing dark inside the enhanced region; (*d*) long-axis LGE imaging; (*e*) LV wall segmentation; (*f*) reconstructed LV geometry, coloured by MI extent (1: 100% MI, 0: healthy myocardium). (Online version in colour.)

In-house developed Matlab (Mathworks, Inc., Natick, MA, USA) code was used to extract the endocardial and epicardial surfaces at early-diastole when the LV pressure is lowest [21]. Short-axis slices from the LV base to apex and three long-axis slices were chosen for manual segmentation, shown in figure 1*a*. Figure 1*b* shows the reconstructed LV geometry. Details of the CMR scans and LV geometry reconstruction are provided in the electronic supplementary material.

In the MI group, CMR scans at 2 days after acute-MI were chosen for model construction. Short- and long-axis late gadolinium enhancement (LGE) images were combined with cine images to define the infarct region, denoted as *U*^MI^ shown in figure 1*c*–*e*. To avoid abrupt change of material properties from *U*^MI^ to functional myocardium, a transition region adjacent to *U*^MI^ was defined within a distance of 10 mm of the infarct boundary, similar to previous work [12]. The reminder of the LV wall was assumed to be unaffected by the infarction, denoted as *U*^un^, or the remote region. A Lagrangian field *M*(**X**) was introduced to describe the extent of the infarction throughout the whole LV geometry *U* as *M* is 1 in the infarct region, 0 in the remote region and linearly decreases from *U*^MI^ towards *U*^un^ in the transition region (figure 1*f*).

In-house developed b-spline deformable registration method was used to estimate regional circumferential myocardial strain from four short-axis slices from the basal plane to mid-ventricle; each slice was divided into six regions according to the AHA 17-segments definition [35]. LV volumes at end-diastole and end-systole were calculated using corresponding cine images. For each LV model, the measurements from one scan consisted of 24 regional circumferential strains and the LV cavity volume. Because it is difficult to acquire myofibre architecture *in vivo*, a rule-based myocardial fibre generation method [11] was used to describe the fibre and sheet orientations of the myocardium. Myofibre rotates from −60° to 60° from endocardium to epicardium, and the fibre along the sheet direction rotates from −45° to 45°. Because end-diastolic pressure in MI patients is usually higher (10–25 mmHg) than healthy subjects (5–10 mmHg) [36], a population-based end-diastolic blood pressure of 8 mmHg was assumed for the healthy group, and 16 mmHg was assumed for the MI group as in [12]. The LV systolic blood pressure (SBP) was approximated by the cuff-measured systolic pressure taken just before the CMR scans.

#### 2.2.2. Myocardial mechanics

The IB/FE method [13,14] is employed to model the ventricular dynamics at end-diastole and end-systole (details can be found in the electronic supplementary material). The myocardial stress tensor (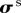) is modelled as the summation of the active (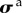) and passive (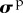) stresses, i.e.2.1
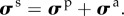
The myofibre stress is2.2
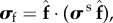
where 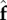 is the normalized myofibre direction in the current configuration. 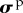 is the passive response described using an invariant-based Holzapfel–Ogden Law [37],2.3
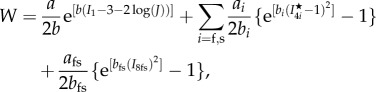
in which *a*, *b*, *a*_f_, *b*_f_, *a*_s_, *b*_s_, *a*_fs_ and *b*_fs_ are eight unknown parameters, 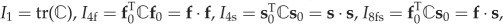, **f**_0_ and **s**_0_ are the initial fibre and sheet directions, and **f** and **s** are the current fibre and sheet directions. 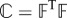 is the right Cauchy–Green deformation tensor, in which 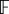 is the deformation gradient. We assume myofibre can only bear load when taut, thus 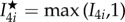. From (2.3), we derive2.4
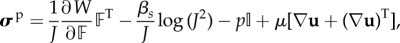
where *β*_s_ = 1.0 × 10^5^ Pa is the bulk modulus, *p* is the Eulerian pressure, *μ* = 4 *cP* is the viscosity, **u** is the Eulerian velocity and 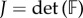. (*β*_s_/*J*)log(*J*^2^) is used to enforce the incompressibility of the immersed solid in addition to the system-wide incompressibility condition 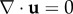 [14].

The active stress is computed as2.5
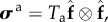
where *T*_a_ is the active tension computed from2.6
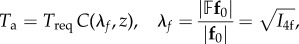
where *T*_req_ is the active tension generated by the myocardium when λ_*f*_ = 1, i.e. *T*_req_ is the minimum value required to meet the pumping demand at the time of imaging. If the innate ability of the myocyte to contract under the maximum activation is denoted as *T*_max_, which may be measured through stress CMR, then the difference between *T*_max_ and *T*_req_ reflects the contractility reserve of the myocardium. *C*(λ_*f*_, *z*) is the effects of myofilament kinetics described by Niederer *et al.* [38] as2.7
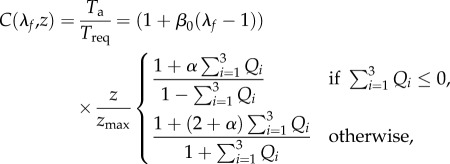
where *z* is the available fraction of actin binding sites that is dependent on the intracellular calcium transient, *z*_max_ is the maximum fraction of actin binding sites at a given λ_*f*_, *β*_0_ is a constant and *α* is a measure of the curvature of the force–velocity relationship, *Q*_*i*_(*i* = 1, 2, 3) are calculated from2.8
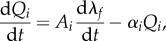
where *A*_*i*_ and *α*_*i*_ are constants. A simple model of intracellular calcium dynamics [39] is used to trigger the myocardial contraction simultaneously. In our simulations, *T*_req_ is inversely estimated to model the patient-specific systolic LV dynamics, other parameters (*β*_0_, *α*, *A*_*i*_, *α*_*i*_, etc.) involved in active tension generation are adopted from Niederer *et al.* [38]. To evaluate systolic myofilament kinetics, we also assess the value of *C*(λ_*f*_, *z*) at end-systole, denoted by *C*^s^.

The differences of the passive and active myocardial responses in the remote, transition and the infarct regions are modelled by changing the strain energy function and active stress [12,26]. Previous studies showed that tissue stiffness due to MI scar increases by 50-fold compared with the remote myocardium [12,27]. Given the similar biological process leading to a MI scar, we assume that the MI scar of all patients is 50 times stiffer2.9
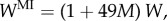
where *M* ∈ [0, 1] takes 0 in the functional myocardium and 1 in the MI region, with a linear transition between them. We further assume the MI tissue does not contract, thus the active tension in the MI heart is2.10
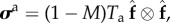
note that partial contractility within MI region is not considered, but assuming the whole MI region is non-contractible. In addition, we adopt the definition used in [21] to define normalized *T*_a_ and *σ*_f_,2.11
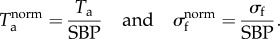


#### 2.2.3. Material parameter identification

**Figure 2.**
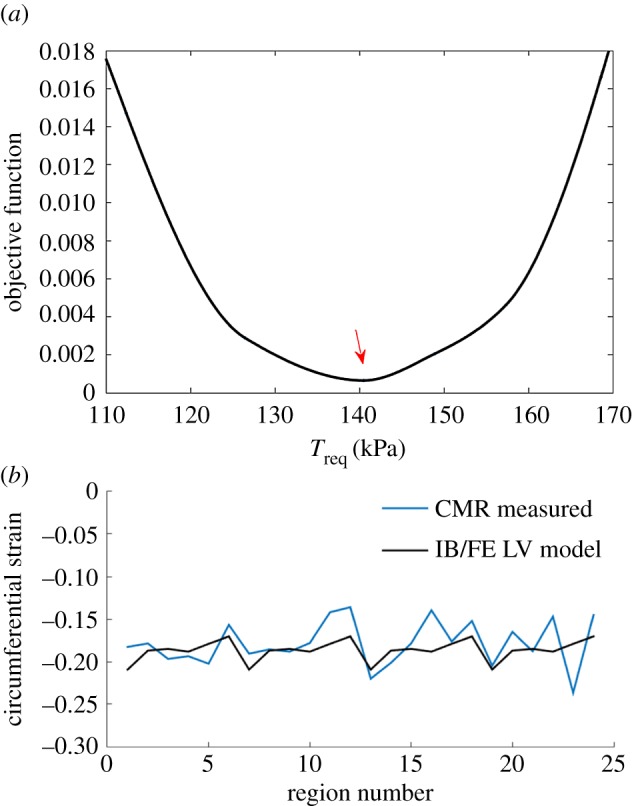
Optimization of *T*_req_ in a healthy heart. (*a*) Finding optimal *T*_req_ by minimizing objective function (equation (2.12)); (*b*) strain comparison between CMR measurements and values from the LV model *T*_req_. The difference in the strain between the measurements and the model prediction is 0.008 ± 0.02. (Online version in colour.)

Material parameters, including *a*, *b*, *a*_f_, *b*_f_, *a*_s_, *b*_s_, *a*_fs_, *b*_fs_ and *T*_req_, are determined so that the simulated LV dynamics are in good agreement with corresponding CMR measurements. Specifically, the passive myocardial parameters are inversely determined from *in vivo* data (LV cavity volume and the regional circumferential strain) using a multi-step optimization scheme [20]. The active parameter *T*_req_ is determined by matching the measured LV end-systolic volume and systolic circumferential strain (CS) of the clinical measurements. All other parameters in the active tension generation model are kept the same as in [12]. The strains are calculated with the reference configuration set at end-diastole. For the healthy subjects, we first inflate the LV model to the end-diastolic pressure and estimate the passive parameters, then initiate the systolic contraction to determine *T*_req_. The objective function is2.12
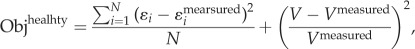
where *ɛ*_*i*_, *V* are the *i*th regional circumferential strain and LV cavity volume from the model, and *ɛ*^mearsured^_*i*_, *V*^measured^ are corresponding measured data. *N* is the total number of the control points. Figure 2*a* shows the optimization of *T*_req_ in a healthy LV model, and figure 2*b* is the strain comparison between the CMR measurements and values from the model predications using the optimized *T*_req_, with good agreement.

For the MI subjects, we only determine the parameters in the remote regions because the MI region is modelled as non-contractile and 50 times stiffer. The passive parameters are determined similarly as for the healthy models, but *T*_req_ in the remote region is determined by minimizing the objective function2.13
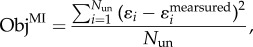
in which *N*_un_ is the number of segmental remote regions. The average strain data from the unaffected region in the MI group is 13 ± 3 out of 24 segments.

Further comparisons on LV cavity volume and strains with *in vivo* CMR measurements are summarized in electronic supplementary material, table S1.

### 2.3. Statistical classification methods

The general characteristics that define the difference between a healthy heart and one that experienced an MI are well known. However, it is less clear whether the functional myocardium and its associated biomechanical factors are sufficient to classify the myocardium from a healthy heart or heart after acute-MI. Here we will apply seven different techniques from multivariate statistics and machine learning to assess the potential of myocardial classification given several biomechanical factors and to identify the methods that yield the highest prediction accuracy.

Our portfolio of methods includes univariate logistic regression, multivariate logistic regression with several predictors and the *k*-nearest neighbours classifier (KNN). Furthermore, linear discriminant analysis (LDA) will be applied, as well as sparse logistic regression with L1 regularization (Lasso). We will evaluate two tree-based methods that exploit the change of entropy in the data, as defined in electronic supplementary material, eqn. (S13). This includes decision trees trained with the C5.0 algorithm, and random forests based on bagging with feature sub-selection. Finally, we will apply a non-parametric Bayesian approach with Gaussian processes and automatic relevance determination (GP-ARD). All these methods are described in more detail in electronic supplementary material, §S0.5.

Another important topic is the identification of relevant factors that exhibit the greatest influence on the classification outcome. In this study, we attempt to find those biomechanical factors that possess the strongest explanatory power for the differentiation of myocardial contractile function from a healthy heart or a heart with acute-MI. Several of the previously mentioned methods provide a measure of factor importance, including the Lasso, Decision-Trees, Random Forests and GP-ARD. We will combine the importance measure of these methods into a single-ranked relevance score to identify the biomechanical factors with the highest explanatory power. The results for both studies are presented in §§3.4 and 3.5.

#### 2.3.1. Method evaluation

We assess the accuracy of correctly predicting myocardial contractile function from either a healthy or MI heart in terms of the true positive (TP), true negative (TN), false positive (FP) and false negative (FN) counts. Positive labels refer to MI heart; negative labels refer to healthy heart. Hence, a *true positive* is the correct identification of an MI heart, a *true negative* is the correct identification of a healthy heart, a *false positive* is the misclassification of a healthy heart as an MI heart and a *false negative* is the misclassification of an MI heart as a healthy heart. These counts are obtained out of sample with leave-one-out-cross validation (LOOCV). With LOOCV, a method predicts the class for one observation *i* ∈ {1, …, *n*} at a time based on the training set of the remaining (*n* − 1) observations, where *n* is the total number of observations in the labelled dataset. From the TP, TN, FP and FN counts, we compute the sensitivity,^1^ specificity^2^ and the total misclassification error^3^ to assess the classification accuracy of a method.

We plot, for all methods included in our study, the sensitivity against the complementary specificity (i.e. 1 minus the specificity). The results will be shown later in figure 10. The convex hull of theses scores presents the receiver operating characteristic (ROC) curve of the ensemble of classifiers we have trained.^4^ By numerical integration, we obtain the area under the ROC curve (AUROC) as an overall indication of the classification performance. An AUROC value of 0.5 indicates random expectation, whereas the maximum value of 1 gives perfect predictive accuracy.

For the non-parametric Bayesian approach, the regularization parameters are optimized by maximizing the marginal likelihood of the training data. For the non-Bayesian approaches, the regularization parameters are obtained based on LOOCV. It is important to note that the out-of-sample data used for evaluating the classification performance must not be used for tuning regularization parameters, to avoid an overoptimistic bias. We, therefore, need two nested LOOCV schemes, one for regularization parameter tuning, the other for method evaluation. This is best illustrated with an example. Considering four data points {1, 2, 3, 4}. For method evaluation, we use an outer LOOCV scheme as follows: training on {1, 2, 3}, evaluating on {4}; training on {1, 2, 4}, evaluating on {3}; training on {1, 3, 4}, evaluating on {2}; training on {2, 3, 4}, evaluating on {1}. For each training set, we use an inner LOOCV scheme for regularization parameter tuning. So for the first fold, {1, 2, 3}, we have: training on {1, 2}, regularization parameter tuning on {3}; training on {1, 3}, regularization parameter tuning on {2}; and training on {2, 3}, regularization parameter tuning on {1}.

## 3. Results

### 3.1. Characteristics of subjects

**Figure 3.**
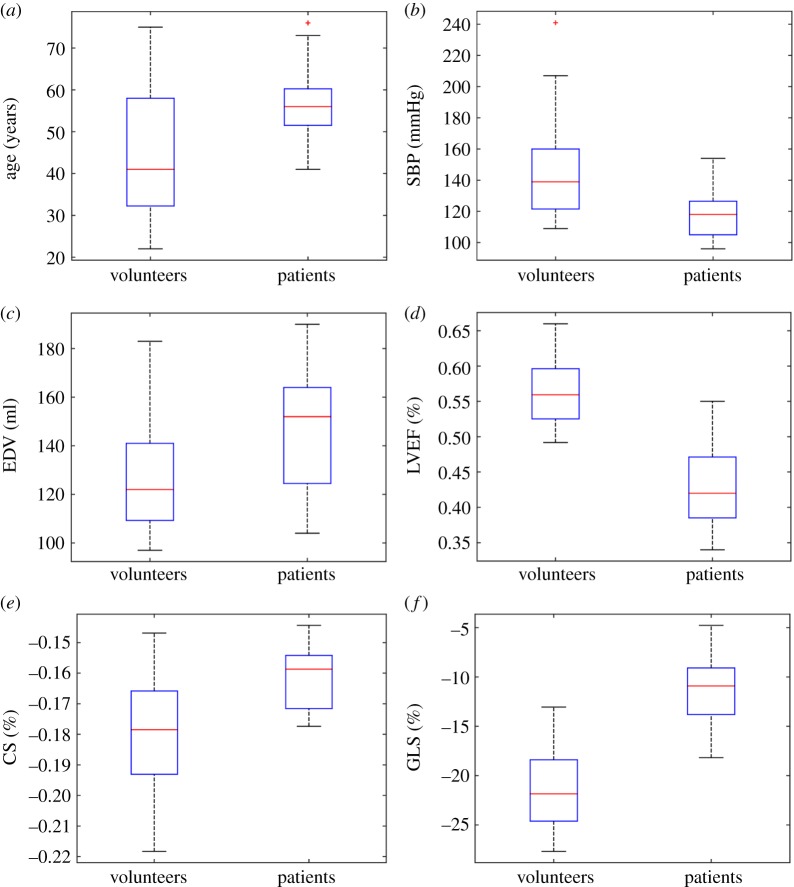
Distributions of age (*a*), SBP (*b*), EDV (*c*), LV [i.e. LVEF] EF (*d*), CS (*e*) and GLS (*f*) in the healthy controls and MI patients at baseline. (Online version in colour.)

The CMR findings of the MI group and the healthy group are summarized in table 1. Distributions of age, end-diastolic volume (EDV), SBP, LVEF and CS are shown in figure 3.

**Table 1.** Basic characteristics of healthy controls and MI patients. *p*-Value is from Student's *t*-test. CS, systolic circumferential strain; GLS, global longitudinal strain.

characteristics	patients	healthy controls	*p*-value
age (year)	57.2 ± 10	44.5 ± 15.4	0.02
sex (male : female)	9 : 2	16 : 11	
systolic blood pressure (mmHg)	118.6 ± 16.4	144.6 ± 31.2	0.01
diastolic blood pressure (mmHg)	73 ± 14	83 ± 15	0.09
LV EF (%)	43 ± 6	57 ± 5	≈0
LV EDV (ml)	145.5 ± 28	127 ± 21	0.02
LV ESV (ml)	83 ± 21	55 ± 14	≈0
GLS (%)	−11.5 ± 3.9	−21.4 ± 4.1	≈0
CS (%)	−0.16 ± 0.01	−0.18 ± 0.02	0.005
infarct size (% LV volume)	39 ± 6.0	—	
microvascular obstruction (% LV mass)	10.6 ± 5	—

### 3.2. Left ventricle mechanics

**Figure 4.**
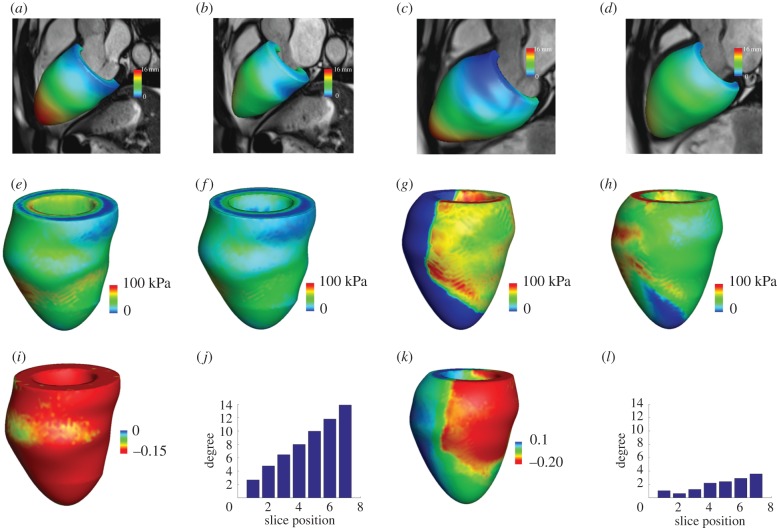
Examples of mechanical modelling of LV dynamics from a healthy control and a MI patient. (*a*) Deformed LV geometry at end-diastole and end-systole (*b*) from a healthy volunteer; (*c*) deformed LV geometry at end-diastole and at end-systole (*d*) from a MI patient; (*e*) distributions of systolic active tension and (*f*) myofibre stress from the healthy volunteer; (*g*) distributions of systolic active tension and (*h*) myofibre stress from the MI patient; (*i*) systolic myofibre strain distribution and (*j*) twist degree from LV base to apex from the healthy volunteer; (*k*) systolic myofibre strain distribution and (*l*) twist degree from LV base to apex from the MI patient. (Online version in colour.)

Figure 4 shows examples of the mechanical modelling of LV dynamics in a healthy subject and a MI patient. Figure 4*a*,*b* is the simulated LV geometries from the healthy control at end-diastole and end-systole, superimposed on CMR cine images, and figure 4*c*,*d* is from the MI model. Figure 4*e*,*f* is the distributions of systolic active tension *T*_a_ and myofibre stress *σ*_f_ in the healthy subject. Since there is no active contraction in the MI region, the MI region is over-stretched to bear the systolic pressure, as shown in figure 4*k*, in which the myofibre strain is positive (in blue), and the remote myocardium region is shortening. The distribution of myofibre stress *σ*_f_ is more homogeneous in the healthy heart compared with the MI heart. Similar pattern is shown in myofibre strain in figure 4*i*,*k*. Figure 4*j*,*l* shows the LV twist relative to the LV base, which linearly increases towards the apex with a maximum apical rotation of around 14° in the healthy model, but only 4° in the MI model.

**Figure 5.**
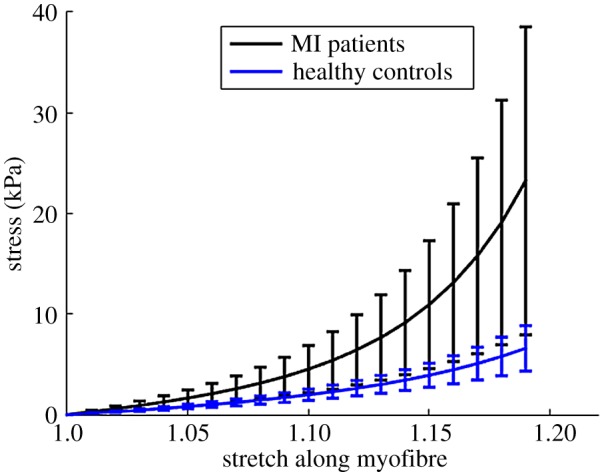
Average passive stiffness along myofibres in healthy controls and MI patients. Error bar is the standard deviation. (Online version in colour.)

Table 2 summarizes the average passive parameters of all the healthy and MI subjects. Figure 5 plots the average passive stiffness along the myofibre direction, and shows that the passive myofibre stiffness in the remote regions of the MI patients is much stiffer than the controls. This is a consequence of the myocardium adaptivity to MI and simply modelled using a higher end-diastolic pressure for the MI patients [36]. This increase corresponds with a stiffer myocardium for a given deformation [20].

**Table 2.** Average passive material parameters.

	*a* (kPa)	*b*	*a*_f_ (kPa)	*b*_f_	*a*_s_ (kPa)	*b*_s_	*a*_fs_ (kPa)	*b*_fs_
controls	0.18 ± 0.1	2.6 ± 0.8	3.34 ± 0.94	2.73 ± 1.06	0.69 ± 0.26	1.11 ± 0.40	0.31 ± 0.18	2.58 ± 0.71
MI subjects	0.09 ± 0.04	4.05 ± 1.6	6.8 ± 3.25	5.53 ± 1.84	1.5 ± 0.66	1.93 ± 0.78	0.16 ± 0.07	4.22 ± 1.7

**Figure 6.**
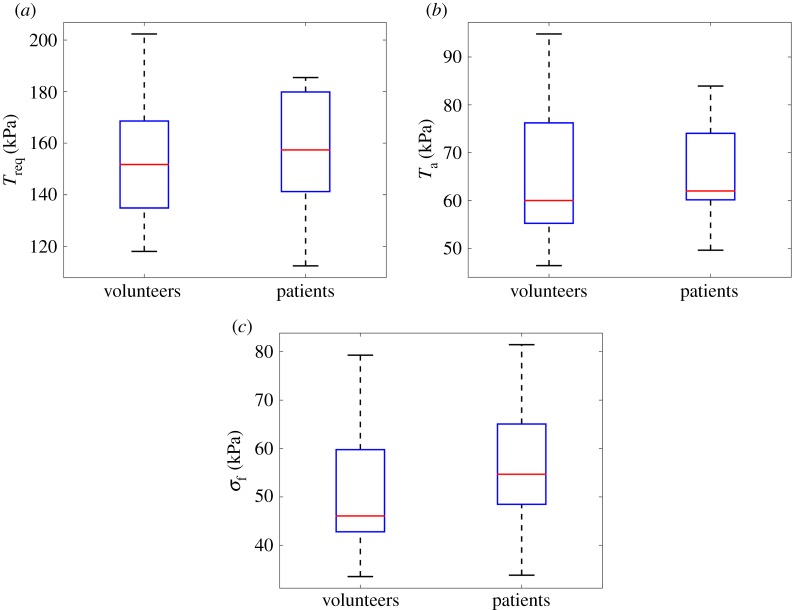
Comparisons of *T*_req_ (*a*), *T*_a_ (*b*) and *σ*_f_ (*c*) between the healthy volunteers and the MI patients. (Online version in colour.)

**Figure 7.**
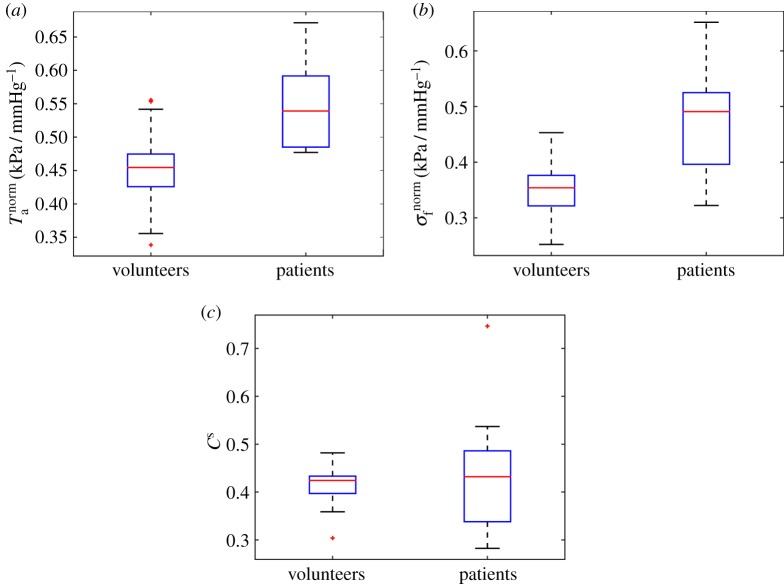
Distributions of *T*^norm^_a_ (*a*), *σ*^norm^_f_ (*b*) and *C*^s^ (*c*) between healthy volunteers and MI patients. (Online version in colour.)

The average *T*_req_ in the healthy group is 157 ± 25 kPa, and 156 ± 27 kPa in the MI group, as shown in figure 6*a*. No significant difference is found in *T*_req_ for the two groups. Similar results are found for *T*_a_ and systolic myofibre stress *σ*_f_ as shown in figure 6*b*,*c*. However, the average *T*^norm^_a_ in the healthy group is much lower than the MI group (0.45 ± 0.06 kPa mmHg^−1^—healthy versus 0.55 ± 0.07 kPa mmHg^−1^—MI, *p* < 0.01). Similar trend is shown in *σ*^norm^_f_ (0.35 ± 0.05 kPa mmHg^−1^—health versus 0.47 ± 0.09 kPa mmHg^−1^— MI, *p* < 0.01). The systolic myofilament kinetics *C*^s^ is 0.42 ± 0.04 in healthy group, which is slightly lower than 0.44 ± 0.13, *p* = 0.50 in the MI group. Figure 7 shows the distributions of *T*^norm^_a_, *σ*^norm^_f_ and *C*^s^. Interestingly, even with a wider range of SBP, the standard deviations of *T*^norm^_a_, *σ*^norm^_f_ and *C*^s^ in the healthy group are much smaller compared with the MI group.

From the biomechanical models, we choose *T*_a_, *T*_req_, *T*^norm^_a_, *σ*_f_ and *C*^s^ as potential biomarkers for myocardial contractile function, but not passive stiffness and diastolic stress because of uncertainties in assumed end-diastolic pressure. A linear correlation analysis is further carried out to decide which features should be included in statistical feature classification and summarized in the electronic supplementary material. The criteria are that if two features are highly correlated in both the healthy and the MI groups, and further related to other features and CMR measurements in a similar way, then the more reliable feature will be selected. For example, *T*_a_ and *σ*_f_ are generally inter-related through the myocardial mechanical model, see equation (2.1), as is also confirmed by the linear correlation analysis. Furthermore, *T*_a_ and *σ*_f_ relate to other features in a similar way, but part of *σ*_f_ is dependent on the passive stress (equations (2.1) and (2.2)). Therefore, *T*_a_ is selected. Although *C*^s^ is correlated to *T*_a_ in both groups, they relate to CMR measurements in different ways; therefore, *C*^s^ is included. Similarly, for *T*_req_ and *T*^norm^_a_. We also include EDV and SBP from CMR measurements because both are inputs for modelling the LV contraction, but not EF, systolic strain and end-systolic volume, which can be reproduced from the LV models. In summary, the selected features for classification of myocardial contractile function between the healthy group and the STEMI group are: SBP, EDV, *T*_a_, *T*^norm^_a_, *T*_req_ and *C*^s^.

### 3.3. Datasets

Three datasets, based on the analysis in §3.2, are evaluated, which differ in the selected biomechanical factors that serve as predictors: the first dataset *D*_1_ includes the factors *T*_req_, *T*_a_, SBP, EDV, *C*^s^ and *T*^norm^_a_. The second set denoted with *D*_2_ lacks the ratios. The third set *D*_3_ includes *T*_a_, EDV, *C*^s^ and *T*^norm^_a_ but not SBP and *T*_req_, whose effects are included in *T*^norm^_a_ and *C*^s^, respectively.

### 3.4. Factor importance

**Figure 8.**
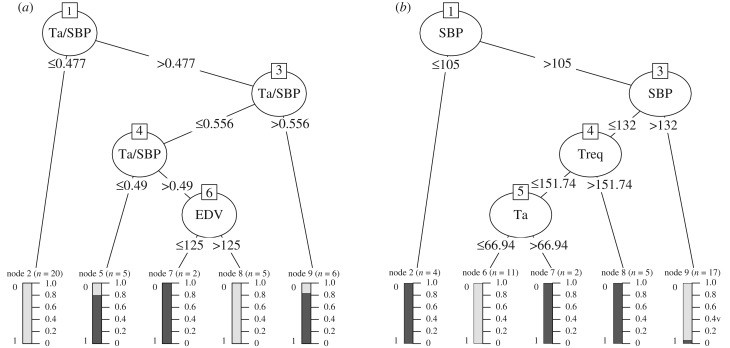
(*a*,*b*) C5.0 Decision-Trees for the different datasets D_1_ (*a*) and D_2_ (*b*). Note that only the first tree for each dataset is shown from an ensemble of boosting trees. The decision tree is the same for dataset D_3_ as dataset D_1_ because the same features are selected for the splits, thus not shown here. The selected C5.0 parameters for tree construction are based on the error rate evaluation in electronic supplementary material, figure S7.

In this section, we discuss the relevance or importance of the various explanatory variables included in the three datasets. For Lasso, the importance measure of an explanatory variable is given by the absolute value of the average regression coefficient associated with that variable. For GP-ARD, the importance measure is expressed as the inverted and normalized length scale associated with the corresponding explanatory variable. The larger the length scale, the larger the change of the corresponding variable has to be to have any effect on the output. For the extreme case of an infinite length scale, any finite change in the corresponding explanatory variable has no effect on the output, and this variable has therefore effectively been switched off. The Decision-Tree provides (figure 8) the usage metric to quantify the importance of an explanatory variable, showing the percentage of times the respective explanatory variable has been selected by the C5.0 algorithm to build the tree. For the Random Forest, the importance measure is the mean decrease in classification accuracy incurred when excluding an explanatory variable from the training set.

**Figure 9.**
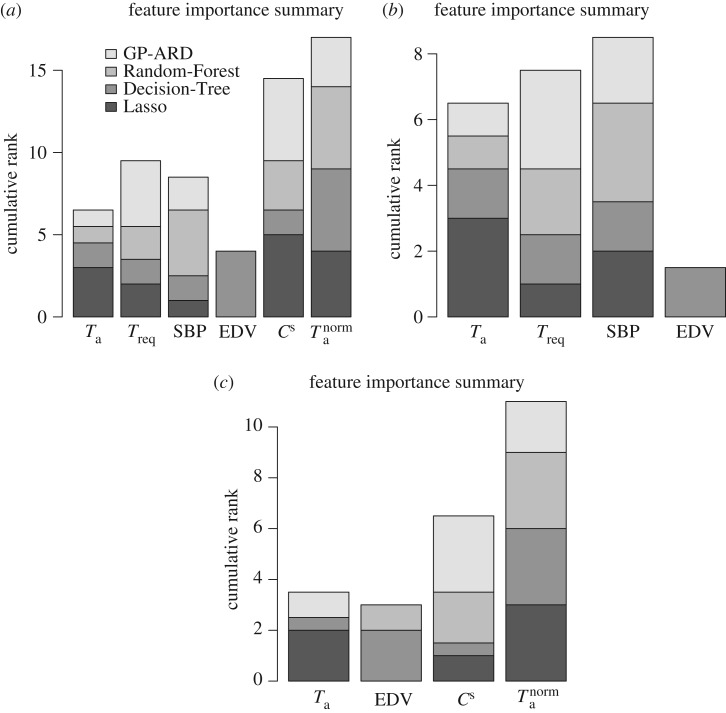
(*a–c*) Summary of feature relevance scores, obtained with different statistical/machine learning methods. The features are separately ranked in ascending order of importance, taken from the set {0, 1, …, *p* − 1}, where *p* is the total number of features. The ranks are accumulated over all methods shown in the legend. These methods are represented by different grey shades in the bars. A higher rank indicates a more important feature. The three panels correspond to the three datasets used in our study; from left to right: *D*_1_, *D*_2_ and *D*_3_.

**Figure 10.**
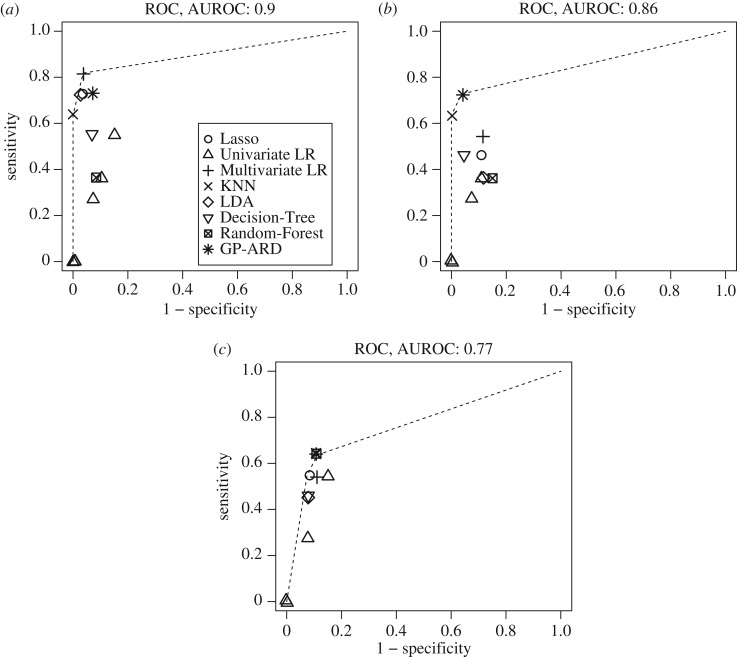
(*a–c*) Summary of classification accuracy for different methods and three datasets *D*_1_, *D*_2_ and *D*_3_ (columns). The area under the ROC curve (AUROC) is calculated from the area under the convex hull (dashed lines) that connects the best performing methods. The specificity and sensitivity score for each method was estimated with LOOCV. Each of the symbols for the univariate logistic regression (univariate LR) corresponds to one feature used as predictor.

For each of these methods, we rank the explanatory variables, and combine the results in an accumulative rank score, which serves as an indicator for the overall factor relevance. These accumulative scores are shown in figure 9, which shows the cumulative ranks based on the importance from 0 to (*p* − 1), where (*p* − 1) is the highest rank. The height of the bars in figure 9 provides a global indication of the association of the respective input variables with the output, or to paraphrase this: the higher the bar in figure 9, the greater is the relevance of the corresponding factor for predicting the classification outcome. It is seen that, overall, the two ratios *C*^s^ and *T*^norm^_a_ have the strongest explanatory power in predicting myocardial contractile function from healthy or MI hearts.^5^ However, our classification results, discussed below and succinctly summarized in figure 10, show that the best performance is achieved when all the available factors are included in the set of predictors. Hence, no individual factor is completely irrelevant.

### 3.5. Classification performance

We have applied the classification methods mentioned in §2.3, and described in more detail in electronic supplementary material, §S0.5, to the three datasets described in §3.3. The results are shown in table 4 and in figure 10. Table 4 shows the sensitivity, specificity and overall misclassification error. The overall method ranking is shown in the last row of table 4 and is based on the sum of misclassification errors over all datasets. Given these scores, KNN and GP-ARD are the preferred methods. Figure 10 shows the sensitivity–specificity score pairs obtained with the different statistical/machine learning methods, for the three datasets described in §3.3. As described in §2.3.1, the convex hull of these scores, indicated by a dashed line, defines the ROC curve that connects the best methods. Methods that lie below this ROC curve are suboptimal. It is seen that none of the statistical/machine learning methods is optimal for all three datasets. However, KNN and GP-ARD are optimal for two datasets, and close to optimal for the third. In agreement with table 4, they can thus be regarded as the best methods overall. Note, in particular, that the univariate methods either clearly fall below the convex hull, or give the trivial score pair of both sensitivity and complementary specificity equal to zero (which is obtained when patients are always classified as healthy). This finding confirms the need for multivariate methods. As discussed in §2.3.1, the ROC curve corresponds to a combination of the best-performing methods, and the area under the ROC curve (AUROC) is a measure of the overall classification performance. Our study indicates that the AUROC score depends on the dataset and varies between 0.77 and 0.9. This is considerably better than random expectation (0.5) and, in the latter case, close to optimal prediction (1.0).

### 3.6. Associations between biomechanical factors at baseline and left ventricle function atsix-month follow-up

Section 3.4 shows that *T*^norm^_a_ and *C*^s^ are the strongest explanatory factors in predicting myocardial contractile function between healthy subjects and MI patients, followed by *T*_req_. Therefore, in this section, the association between *T*^norm^_a_, *C*^s^, *T*_req_ and LV pump function recovery at six months is further analysed. Figure 12 shows the relationship between LV pump function recovery at six months and *T*_req_, *C*^s^ at baseline in the MI group. It can be seen that a strong linear relationship between *T*_req_, *C*^s^ at baseline and LVEF changes at six months. With a lower *T*_req_, the MI patient could have a better LVEF recovery after six months. Thus, we hypothesize that for acute-MI patients with a similar calcium handling dynamics, a lower *T*_req_ suggests a potential to further increase the contractile function, without going into de-compensated states, compared with cases when the myocardial contractility already reaches the maximum. Since the maximum myocardial contractility is limited, less usage of the contractile reserve will give the heart more resilience in the longer term. We also observe that *C*^s^ is positively related to LV EF changes at six months. Since *C*^s^ is associated with the myofilament kinetics, this presumably suggests there are more binding sites available at systole for generating the active tension. However, no correlations are found between *T*_a_, *T*^norm^_a_, *σ*_f_, *σ*^norm^_f_ and LV function at six-month follow-up, which are summarized in table 3.

**Table 3.** Associations between global measures of LV systolic function early post-MI and surrogate outcomes of LV function at six-month follow-up. A *p*-value ≤0.05 is taken as statistically significant (in bold).

	change in LVEF after six months			GLS after six months		
baseline	coefficient	95% CI	*p*-value	coefficient	95% CI	*p*-value
LVEF (%)	−0.26	[−0.74, 0.41]	0.45	−0.43	[−0.82, 0.23]	0.19
GLS (%)	−0.05	[−0.63, 0.57]	0.90	0.73	[0.22, 0.92]	0.01
CS (%)	0.43	[−0.23, 0.82 ]	0.19	0.12	[−0.52, 0.67]	0.73
*T*_req_ (kPa)	**−0.79**	[−0.94, −0.37]	**0.003**	0.27	[−0.40, 0.75]	0.43
*T*_a_ (kPa)	0.33	[−0.33, 0.78]	0.31	−0.13	[−0.68, 0.51]	0.70
*σ*_f_ (kPa)	0.46	[−0.19, 0.83]	0.15	−0.14	[−0.68, 0.50]	0.68
*T*^norm^_a_ (kPa mmHg^−1^)	0.24	[−0.42, 0.73 ]	0.48	−0.08	[−0.65, 0.55]	0.82
*σ*^norm^_f_ (kPa mmHg^−1^)	0.48	[−0.17, 0.84]	0.13	−0.13	[−0.68, 0.51]	0.70
*C*^s^	**0.70**	[0.16, 0.91]	**0.02**	−0.26	[−0.74, 0.41]	0.45

## 4. Discussion

### 4.1. The LV mechanical modelling

The major determinant of long-term survival after an acute-MI is the efficiency of LV pump function. Despite decades of research, the pathophysiology and disease mechanisms of the failing heart remain incompletely understood, engendering a knowledge gap for the development of new therapies. It is believed that novel mathematical tools are required to solve those fundamental patho-physiological questions. In this study, we have modelled the LV dynamics at end-diastole and end-systole in healthy controls and in patients with recent STEMI. The computational contracting LV models are based on *in vivo* CMR data. Using this approach, we inversely determine the passive parameters and contractility for all the LV models. We have shown for the first time that, compared with healthy controls, patients with recent STEMI exhibit increased active tension, i.e. increased contractility and increased active tension, when normalized by SBP.

It is challenging to estimate myocardial passive stiffness inversely, especially with limited measurements *in vivo*. Our previous study [20] showed that although the eight constitutive parameters from the Holzapfel–Ogden Law (equation (2.3)) cannot be uniquely estimated inversely, the mechanical response along myofibre direction for a functional myocardium can be robustly obtained. One of the main limitations in estimating the passive myocardial parameters in this study is the assumed population-based end-diastolic pressure, and the MI group has a higher end-diastolic pressure (16 mmHg) compared to the healthy control groups (8 mmHg). The end-diastolic pressure is one of the key input in the parameter estimation, and a higher pressure is associated with a stiffer myocardium. Without knowing the actual end-diastolic pressure for the MI and healthy groups, the quantitative change in the myocardial stiffness may be subject to some uncertainty. However, previous studies [17] and our own experiments show that the passive stiffness uncertainty arising from end-diastolic pressure has few effects on the contractility (*T*_req_) estimation because of the matched EDV and the strain data.

The patient-dependent myocardial systolic contractility in our simulations is controlled by the parameter (*T*_req_), all other parameters of the active tension generation are kept fixed. This allows us to match measured LV dynamics in end-systole, and avoid the complexity of determining many parameters in the electromechanical models from different functional and structural scales. This approach has been widely applied when mathematically simulating LV systolic dynamics [12,21,26,27]. The average required myocardial contractility for the control group is 157 ± 25 kPa in our study, which is comparable with the reported value by Genet *et al.* [21] from five normal human subjects, the peak contractility reported by Arsner *et al.* [29] in one healthy volunteer (139 kPa), and the average contractility reported by Wang *et al.* [40] from six healthy human subjects.

A transition region is defined in MI hearts to avoid abrupt change of material properties from MI to functional myocardium following our previous study [12]. We remark this does not necessarily represent the MI border zone which can also be modelled with partial contractility as in [41]. We did not attempt to model the detailed border zone region effects in this study as we could not extract the border zone and its viability from the CMR images. Thus, we adopted a simplified approach to only consider two regions in the MI hearts: the functional myocardium and non-contractile MI region. Future work to accurately define the border zone and the material properties within and around the scar based on new imaging protocols and detailed experimental tests is required. Indeed, mathematical models have been developed which can map the myocardial viability from LGE images that provide partial contraction inside MI [42].

It has been found that myocardial mechanics are dependent on the underlying fibre architecture [11]. However, because of the difficulties of acquiring detailed subject-specific fibre architecture *in vivo*, such fibre structures are usually not included in computational LV models. Instead, most models, this study included, employ rule-based fibres [7,26,42]. To assess the sensitivity of the results due to small changes of fibre architecture, we further increased the myofibre angle by 10% in a healthy heart, that was, −66° to 66°, compared with the original myofibre rotation from −60° to 60°. We found that *T*_req_ was decreased by 6%, and *T*_a_ was slightly reduced by (≈ 1.8%).

We are aware that our current mechanical model is still necessarily simplified, in particular, with one chamber and the LV dynamics are simulated at two time points only (end-diastole and end-systole). Moreover, a simplified intracellular transient is used to trigger active contraction simultaneously, due to lack of parametrization in cellular level, *C*^s^ may only represent an ‘apparent’ myofilament kinetics under same intracellular calcium dynamics.

However, this is a starting point towards clinical translation in terms of real time prediction, and our combined mechanical, statistical and MRI study already show promising value in evaluating myocardial functions between the healthy subjects and MI patients.

### 4.2. Statistical classification

Our ultimate aim is to build a classifier (case versus control) based on *in vivo* imaging data described in the electronic supplementary material. However, working directly on the images, e.g. representing them as grey-level pixel vectors and building a classifier in this high-dimensional space, leads to the well-known curse-of-dimensionality problem [43]. Standard approaches, therefore, carry out a dimension reduction first. In the simplest case, this can be done with principal component analysis. More advanced methods aim to improve dimension reduction by identifying low dimensional submanifolds of the high-dimensional configuration space that contain the relevant information related to the classification problem at hand. Our present research can be seen as an extension of previous work whereby the low-dimensional configuration space is directly spanned by the parameters of an explicit biomechanical model. This is model-based rather than purely data-driven dimension reduction, with the advantage that for a reliable and accurate model, the reduced configuration space is *a priori* highly likely to contain physiologically relevant information.

We have worked in the biomechanical parameter space with a variety of methodological tools. We have started with a simple univariate analysis, followed by a multivariate approach, where we have used the latter to evaluate the relative explanatory relevance of the various biomechanical parameters with respect to the classification task and to compare the performance improvement obtained with several multivariate classification methods over the univariate approach.

In the univariate approach, by simply comparing the different biomechanical parameters, several parameters are identified for classifying myocardial contractile function from either a healthy heart or a MI heart, including SBP, EDV, *T*^norm^_a_, *T*_req_, *C*^s^ and *T*_a_. However, the simplified comparison, such as using the S student *t*-test, has its limitations, as shown in figure 7. Only *T*^norm^_a_ is found to be significantly different between the control group and the MI group. In the second part of the study, we have shown that multivariate statistical methods provide more powerful tools for classifying the difference in myocardial contractile function after acute-MI.

In the second part, we have used several machine learning and computational statistical methods to identify the biomechanical factors that possess the strongest explanatory power for predicting the changes in myocardial contractile function after acute MI. Our study has revealed that *C*^s^ and *T*^norm^_a_ are the most relevant factors for this classification task, but that no individual factor is completely irrelevant. For details, see §3.4.

**Figure 11.**
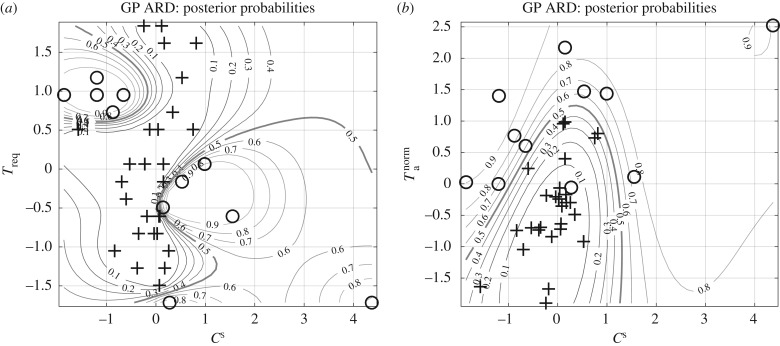
(*a,b*) Posterior probability plots of Gaussian process—automatic relevance determination (ARD). The two plots show the posterior probability contours given the most important features estimated by GP-ARD from fig. S2: *C*^s^, *T*_req_ and *T*^norm^_a_. The plus symbols correspond to healthy volunteers and the circles to patients with MI. The decision boundary of 0.5 is highlighted with a thick grey line.

In the next evaluation step, we have predicted myocardial contractile function between healthy and MI hearts based on the previously mentioned biomechanical factors with eight different methods. Figure 10 and table 4 summarize the prediction performance and the overall best possible performance given all methods. The univariate approach (Univariate LR) shows the poorest performance, as discussed in §3.5. Among the multivariate approaches, KNN and GP-ARD show the best performance (see §3.5 for more details). GP-ARD allows us to visualize the posterior probability of MI heart as a function of the two most important factors; this is shown in figure 11. The plots in this figure illustrate three things. Firstly, it requires more than one factor or variable to separate the data into the two classes. This confirms that a univariate approach is too restrictive, and that a multivariate approach is needed. Secondly, the separation boundaries are nonlinear. This explains why the nonlinear methods, KNN and GP-ARD, show in general a better performance than their linear counterparts. Thirdly, the folding of the decision boundary is very narrow. This explains why the best performance with KNN is achieved with low values of *k*.

**Table 4.** Overview of misclassification error rate and sensitivity/specificity measures for different methods and datasets. A low error, and high sensitivity/specificity indicate better prediction accuracy. The corresponding ROC plot is shown in figure 10. For the univariate logistic regression (Univariate LR), only the prediction with the lowest error is shown. The best scores for each dataset are shown in bold. The last row indicates the method ranks based on the sum of errors from each dataset. The lowest ranks correspond to methods with the highest classification accuracy.

		Lasso	Univariate LR	Multivariate LR	KNN	LDA	Decision-Tree	Random-Forest	GP-ARD
*D*_1_	error	0.11	0.24	**0.079**	0.11	0.11	0.18	0.21	0.13
	specificity	0.96	0.85	0.96	**1**	0.96	0.93	0.96	0.93
	sensitivity	0.73	0.55	**0.82**	0.64	0.73	0.55	0.36	0.73
*D*_2_	error	0.24	0.26	0.21	**0.11**	0.26	0.18	0.29	**0.11**
	specificity	0.89	0.89	0.89	**1**	0.89	0.96	0.85	0.96
	sensitivity	0.45	0.36	0.55	0.64	0.36	0.45	0.36	**0.73**
*D*_3_	error	0.21	0.24	0.21	**0.18**	0.21	0.21	**0.18**	**0.18**
	specificity	**0.93**	0.85	0.89	0.89	**0.93**	**0.93**	**0.93**	0.89
	sensitivity	0.45	0.55	0.55	**0.64**	0.45	0.45	0.55	**0.64**
	rank (error sum)	4 (0.56)	8 (0.74)	3 (0.49)	1 (0.4)	6 (0.58)	5 (0.57)	7 (0.68)	2 (0.42)

The overall predictive accuracy given all methods can be summarized with the AUROC score, which is displayed in figure 10 for each dataset. As it was previously mentioned, the overall accuracy improves from 0.86 to 0.9 when *C*^s^ and *T*^norm^_a_ are added to the single factors present in dataset *D*_2_. This AUROC value is considerably larger than random expectation (0.5) and quite close to perfect prediction (1.0). To rule out that *C*^s^ and *T*^norm^_a_ are for themselves sufficient predictors for the two heart conditions, we excluded *T*_req_ and SBP and found a significant drop in the AUROC value to 0.77. This implies a synergy, i.e. interaction effect, of the involved factors. Hence all the available factors present in *D*_1_ are important myocardial contractile function indicators that should be used in conjunction to differentiate myocardial contractile function between healthy and MI hearts.

### 4.3. Clinical implication

**Figure 12.**
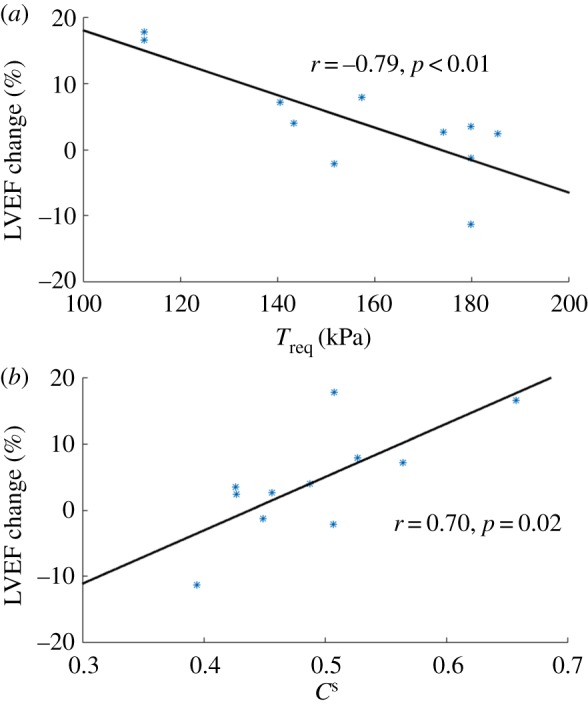
(*a,b*) Linear correlation analysis between *T*_req_, *C*^s^ and LV function recovery at six months after acute-MI. (Online version in colour.)

The estimated overall myocardial contractility *T*_req_, *T*^norm^_a_ and *C*^s^, as suggested in the classification part, might potentially be biomarkers for risk stratification of MI patients. However, since our study has a limited sample size with selected patients, it is not immediately obvious which, if any, of the biomechanical parameters might have clinical prognostic value, and further research is warranted. The linear correlation analysis (figure 12) might suggest that *T*_req_ and *C*^s^ could be potentially used to identify MI patients who may have better recovery by using inotropic treatment if *T*_req_ is in a normal range or by reducing *T*_req_ if it is high.

Future prospective studies should investigate the mechanism of how *T*_req_, *C*^s^ and *T*^norm^_a_ link with myocardial contractile function through different pathways. The effects of novel therapies on these biomechanical parameters should also be assessed. Finally, future studies should evaluate whether these to determine the incremental prognostic value of *T*_req_, *C*^s^ and *T*^norm^_a_ to identify individual patients at high risk of heart failure post-MI, over and above standard prognostic markers such as natriuretic peptides. The strategy to achieve such improvements could involve identification of high-risk patient subsets, identification of subsets of patients with viable myocardium who might be expected to respond to therapy, and implementation of more intensive therapy. Future heart failure management and therapies may be targeted to restore the overall LV contractile reserve according to the level of myocardial contractility and its reserve, such as either downregulating of adrenergic signalling to reduce contractility or using inotropic treatments to enhance it.

## 5. Conclusion

Using combined personalized computational cardiac biomechanical modelling and statistics analysis, we have studied systolic LV dynamics for one patient group consisting of 11 acute STEMI patients with no-reflow and a group of healthy control subjects, based on CMR imaging. The passive response and active contractile properties of myocardium are determined by matching the simulated LV dynamics (volume and circumferential segmental strains) to the CMR measurements. We find that, compared with healthy controls, patients with STEMI exhibit increased LV wall active tension when normalized by SBP. This suggests that the functional myocardium in the patients are overcompensating in order to preserve the stroke volume. Different machine learning and multivariate statistical analysis methods are applied to identify the biomechanical factors that possess the explanatory power in terms of the changed myocardial contractile function after STEMI. The individual required contractility (*T*_req_), normalized active tension *T*^norm^_a_ and the systolic myofilament kinetics *C*^s^ are found to have the strongest explanatory power, and the statistical method KNN has shown the best performance for the classification of myocardial contractile function, followed by GP-ARD. We further observe strong correlations between the biomarkers (*T*_req_, *C*^s^) and the changed LVEF at six months from baseline (*r* = − 0.79, *p* < 0.01, *r* = 0.70, *p* = 0.02). We conclude that the patient-specific contractility *T*_req_, the normalized active tension *T*^norm^_a_, and the myofilament kinetics *C*^s^ all have potential clinical values for prognostication on LV contractile status post-MI, their significance merits further study in larger and unselected patient cohorts.

## Supplementary Material

Supplementary Material

## Endnotes

1 The sensitivity, also called the true positive rate (TPR) or recall, is the proportion of correctly identified MI hearts, and is defined as TP/(TP + FN).

2 The specificity, also called the true negative rate (TNR) or 1 minus false positive rate, is the proportion of correctly classified healthy hearts, defined as TN/(TN + FP).

3 The total misclassification error is defined as (FN + FP)/*n* = (FN + FP)/(TP + FP + TN + FN).

4 Consider two classifiers, with true positive rates TRP_1_ and TRP_2_, and with true negative rates TNR_1_ and TNR_2_. Now define a family of new classifiers as follows: with probability λ ∈ [0, 1], they use classifier 1, and with probability (1 − λ), they uses classifier 2. The expected true positive and true negative rates of these classifiers are λTRP_1_ + (1 − λ)TRP_2_ and λTNR_1_ + (1 − λ)TNR_2_, respectively; hence, they lie on a straight line connecting the scores of the two original classifiers, (TRP_1_, TRN_1_) and (TRP_2_, TRN_2_). From the TRP and TNR scores of the ensemble of classifiers, we can thus obtain the convex hull by linear interpolation between the most extreme (TRP, TNR) pairs. This is the ROC curve representing the optimal classifier combinations (figure 10).

5 This can be seen from figure 11*a*. The figure shows the classification boundaries between MI patients and healthy controls in the two-dimensional space spanned by two parameters: *C*^s^ and *T*_req_. The two patient groups can be clearly separated by the nonlinear decision boundaries. However, a successful classification requires information about both values of *C*^s^ and *T*_req_ together. The values for *C*^s^ are distributed across the whole parameter range for both classes; so knowing *C*^s^ alone, without *T*_req_, will not allow successful classification of myocardial contractile function as MI versus control.

## Data accessibility

Electronic supplementary material is provided. The IB/FE simulation platform can be found from https://github.com/IBAMR/IBAMR. Statistical analysis is done using *R* (https://www.r-project.org/).
