# Supplementary material for: Changes and classification in myocardial contractile function in the left ventricle following acute myocardial infarction
Source: J R Soc Interface. 2017 Jul 26;14(132):20170203. doi: 10.1098/rsif.2017.0203 (PMC5550971; doi:10.1098/rsif.2017.0203)
Supplement: Supplementary Material [file rsif20170203supp1.pdf]

Supplementary Material

Changes and classification in myocardial contractile function in the left ventricle following acute myocardial infarction

Hao Gao[1], Andrej Aderhold[1], Kenneth Mangion[2], Xiaoyu Luo[1], Dirk Husmeier[1], and Colin Berry[2]

[1] School of Mathematics and Statistics, University of Glasgow, UK
[2] British Heart Foundation Glasgow Cardiovascular Research Centre, University of Glasgow, UK

S0.1 Mechanical LV Model

Study population and in vivo imaging We performed a prospective, observational CMR cohort study involving serial CMR scans obtained in a single regional cardiac centre between 14 July 2011 and 22 November 2012. Three hundred and forty three STEMI patients provided written informed consent to undergo CMR 2.2 ± 1.9 days and 6 months post-MI. Patients were eligible if they had an indication for primary PCI or thrombolysis for acute STEMI due to a history of symptoms consistent with the acute myocardial ischemia and with supporting changes on the electrocardiogram (ECG) (i.e. ST-segment elevation or new left bundle-branch block). Exclusion criteria represented standard contra-indications to contrast CMR, including a pacemaker and estimated glomerular filtration rate $\leq$ 30 ml/min/1.73 m^2. The study was approved by the National Research Ethics Service, and all participants provided written informed consent. Acute STEMI management followed contemporary guidelines. The ClinicalTrials.gov identifier is NCT02072850.

Eleven STEMI patients with no reflow were chosen (the MI group), and twenty seven volunteers without the history of cardiac diseases were also enrolled as the healthy control group. CMR was performed on a Siemens MAGNETOM Avanto (Erlangen, Germany) 1.5-Tesla scanner with a 12-element phased array cardiac surface coil. Patients and healthy volunteers underwent the same imaging protocol except that the healthy volunteers $<$ 45 years did not receive gadolinium.

The CMR imaging protocol involved steady-state free precession cine imaging, which was used for LV structure and functional assessment, short-axis cine stack of the left ventricle from the base to apex was acquired and consisted of 7 mm thick slices with a 3 mm inter slice gap. Typical imaging parameters were: matrix 180×256, flip angle 80°, TR 3.3 ms, TE 1.2 ms, bandwidth 930Hz/pixel, and voxel size $1.3 \times 1.3 \times 7\,\text{mm}^3$. Cine images were acquired in the three-chamber, horizontal long-axis, and vertical long-axis planes. In the STEMI group, late gadolinium enhancement images covering the entire LV were acquired 10-15 minutes after IV injection of 0.15 mmol/kg of gadoterate meglumine (Gd2+-DOTA, Dotarem, Guebert S.A.) using segmented phase-sensitive inversion recovery (PSIR) turbo fast low-angle shot sequence. Typical imaging parameters were: matrix $= 192 \times 256$, flip angle $= 25^o$, TE $=$ 3.36 ms, bandwidth $=$ 130 Hz/pixel, echo spacing $=$ 8.7ms and trigger pulse $=$ 2. The voxel size was $1.8 \times 1.3 \times 8$ mm^3. Inversion times were individually adjusted to optimize nulling of the apparently normal myocardium (typical values, 200 to 300 ms). The CMR methods and analyses have been previously described in detail [1]. Blood pressure was measured according to standard guidelines using a standard size cuff, and a larger or smaller bladder was used for large or thin arms. We observed that some of these healthy volunteers had an elevated systolic blood pressure (SBP) at the time of the CMR scan. The increased SBP at the time of the CMR scan may have reflected psychological stress or possibly undiagnosed arterial hypertension.

CMR imaging motion correction To correct motion artefacts from CMR scans, segmented endocardial and epicardial boundaries from short-axis images are realigned manually according to long-axis views (one-chamber, left ventricular outflow tract (LVOT), and four-chamber views), so that the ventricular boundaries from short-axis images are in the same position as in long-axis views. Detailed steps are:

- **Step 1**: Endocardial and Epicardial boundaries are segmented manually in short-axis images, represented by the red curves in Fig. S1(a).
- **Step 2**: Intersection lines in which long-axis views meet short-axis images are defined according to CMR scan protocols using the CMR scanner coordinate system, which can be obtained from the header information of DICOM files, represented by the blue lines in Fig. S1(a). The short-axis images will be translated along those intersection lines.
- **Step 3**: Original boundaries are plotted with long-axis views in the CMR scanner coordinate system for manually translation, as shown in Fig. S1(b). Only endocardial boundary is plotted.
- **Step 4**: Ventricular boundaries from short-axis views are translated manually to the boundaries revealed from the long-axis view images (Fig.S1(c)).
- **Step 5**: Boundaries from each short-axis image are corrected in a similar way (Fig. S1(d)).

The motion artefacts as shown in Fig. S1(b) are corrected after the realignment to long-axis view images, as shown in Fig. S1(c). Fig. S1(d) shows the final LV boundaries after motion correction, which are used for LV geometry reconstruction.

IB/FE formulation The immersed boundary (IB) method [2] is employed to model the fluid-structure interaction (FSI) of the ventricular dynamics at end-diastole and end-systole, in which an incompressible ventricle is immersed in a viscous incompressible fluid. The immersed solid are described in a Lagrangian formulation, including the deformation and stresses. The momentum, viscosity, and incompressibility of the fluid is described in an Eulerian formulation. Let Ω denote the physical domain occupied by the FSI system, and let U denote the reference coordinate system attached to the immersed solid, and $\mathbf{X} = (X_1, X_2, X_3) \in U$ is material (Lagrangian) coordinates attached to the immersed solid. Let $\mathbf{x} = (x_1, x_2, x_3) \in \Omega$ denote the fixed Eulerian coordinates. The current physical region of the immersed solid at time t is $\Omega^{\mathrm{s}}(t) = \boldsymbol{\chi}(U, t)$, and the fluid region at time t is $\Omega^{f}(t) = \Omega \backslash \Omega^{\mathrm{s}}(t)$. In brief, the IB/FE description

of the fluid-structure coupled system is given by the following equations [3].

$$\rho\left(\frac{\partial \mathbf{u}}{\partial t}(\mathbf{x},t)+\mathbf{u}(\mathbf{x},t)\cdot\nabla\mathbf{u}(x,t)\right)=-\nabla p(\mathbf{x},t)+\mu\nabla^2\mathbf{u}(\mathbf{x},t)+\mathbf{f}^{\mathrm{s}}(\mathbf{x},t), \tag{S1}$$

$$\nabla\cdot\mathbf{u}(\mathbf{x},t)=0, \tag{S2}$$

$$\begin{aligned}\mathbf{f}^{\mathrm{s}}(\mathbf{x},t)=&\int_U \nabla_{\mathbf{X}}\cdot\mathbb{P}^{\mathrm{s}}(\mathbf{X},t)\,\delta(\mathbf{x}-\boldsymbol{\chi}(\mathbf{X},t))\,\mathrm{d}\mathbf{X}\\ &-\int_{\partial U}\mathbb{P}^{\mathrm{s}}(\mathbf{X},t)\,\mathbf{N}(\mathbf{X})\,\delta(\mathbf{x}-\boldsymbol{\chi}(\mathbf{X},t))\,\mathrm{d}A,\end{aligned} \tag{S3}$$

$$\frac{\partial\boldsymbol{\chi}}{\partial t}(\mathbf{X},t)=\int_\Omega \mathbf{u}(\mathbf{x},t)\,\delta(\mathbf{x}-\boldsymbol{\chi}(\mathbf{X},t))\,\mathrm{d}\mathbf{x}, \tag{S4}$$

in which ρ is the fluid density, μ is the fluid viscosity, $\mathbf{u}$ represents the Eulerian velocity, and p is the Eulerian pressure field. $\delta(\mathbf{x}) = \delta(x_1)\,\delta(x_2)\,\delta(x_3)$ is a smoothed three-dimensional Dirac delta function, $\mathbf{f}^{\mathrm{s}}$ represents the Eulerian force density derived from the first Piola-Kirchoff stress tensor $\mathbb{P}^{\mathrm{s}}$ of the immersed structure. These equations express the conservation of the momentum and mass in the Eulerian form while using a Lagrangian description for the structural deformation and stress tensor of the immersed solid.

The total Cauchy stress for the fluid-structure coupled system is

$$\boldsymbol{\sigma}(\mathbf{x},t)=-p\,\mathbb{I}+\mu\left[\nabla\mathbf{u}+(\nabla\mathbf{u})^T\right]+\begin{cases}\boldsymbol{\sigma}^{\mathrm{s}}(\mathbf{x},t) & \text{for } \mathbf{x}\in\text{immersed solid,}\\ \mathbf{0} & \text{for } \mathbf{x}\in\text{otherwise,}\end{cases} \tag{S5}$$

in which $\boldsymbol{\sigma}^{\mathrm{s}}$ describes the passive elastic and active stress of the myocardium, and it relates to the first Piola-Kirchhoff stress via

$$\boldsymbol{\sigma}^{\mathrm{s}}=J^{-1}\,\mathbb{P}^{\mathrm{s}}\,\mathbb{F}^T, \tag{S6}$$

where $\mathbb{F}=\partial\boldsymbol{\chi}/\partial\mathbf{X}$ is the deformation gradient, and $J=\det(\mathbb{F})$. To use standard C^0 finite element methods for non-linear elasticity, a weak formulation for the structure domain is employed as in Ref [3] by introducing an equivalent weak formulation of $\mathbf{f}^{\mathrm{s}}$, namely

$$\mathbf{f}^{\mathrm{s}}(\mathbf{x},t)=\int_U \mathbf{F}^{\mathrm{s}}(\mathbf{X},t)\delta(\mathbf{x}-\boldsymbol{\chi}(\mathbf{X},t))\mathrm{d}\mathbf{X} \tag{S7}$$

$$\int_U \mathbf{F}^{\mathrm{s}}(\mathbf{X},t)\cdot\mathbf{V}(\mathbf{X})\mathrm{d}\mathbf{X}=-\int_U \mathbb{P}^{\mathrm{s}}(\mathbf{X},t):\nabla_{\mathbf{x}}\mathbf{V}(\mathbf{X})\mathrm{d}\mathbf{X} \tag{S8}$$

where $\mathbf{F}^{\mathrm{s}}$ is the Lagrangian structural force density and $\mathbf{V}(\mathbf{X})$ is an arbitrary Lagrangian test functionals. Eqs. S7-S8 enable us to numerically obtain $\mathbf{f}^{\mathrm{s}}$ through $\mathbf{F}^{\mathrm{s}}$ using a standard Lagrangian finite element scheme. Further details can be found in Ref [3].

Boundary conditions and implementation The axial and circumferential displacements in the basal plane is set to be zero to keep the left ventricle in

place, radial placements in the basal plane is not constrained to allow basal radial expansion. The reminder of the LV wall, including the apex, is left free. Along Ω, a combination of zero normal traction and zero tangential velocity boundary conditions are imposed along $\partial\Omega$. A spatially uniform pressure load is applied to the endocardial surface of the LV model, and linearly ramped to an assumed end-diastolic value and allow the LV wall to expand until achieve its measured end-diastolic volume. Then the pressure is rapidly increased to the end-systolic value, which is approximated by the cuff-measured peak pressure. The intracellular Ca^{2+} is gradually increased to its peak value to induce myocardial active contraction. Computations end when a steady state is reached. A population-based end-diastolic pressure is assumed for the healthy group, which is 8 mmHg, and the end-systolic pressure is approximated by the cuff-measured peak pressure. In the MI group, 16 mmHg is assumed for the end-diastolic pressure, which is higher than the healthy controls, and the end-systolic pressure is approximated from the cuff-measured pressure.

LV models are immersed in a 15 cm×15 cm×20 cm fluid box which is discretized with a 96×96×128 regular Cartesian grid, corresponding to a grid spacing $\Delta x = 0.156$ cm. Our previous study of LV dynamics showed that a Cartesian grid spacing of $\Delta x = 0.156$ cm yielded grid-converged results, thus in this study we do not perform a new grid convergence study. A standard four-point regularized version of the delta function is used to approximate the singular delta function kernel in the Lagrangian-Eulerian interaction functions (S3) and (S4). Dynamically generated Gaussian quadrature rules are used to ensure a density of at least two quadrature points per Cartesian grid width. A time step size of 1.22e-4 s is used in diastole and a much smaller step size (3.0e-5s) is used in systole due to the explicit time stepping scheme employed in our numerical implementation. Further details of the numerical scheme can be found in Ref [3].

All simulations are implemented within the open-source IBAMR software, which is an adaptive and distributed-memory parallel realization of the IB methods, it uses other open-source libraries, including SAMRAI, PETSc and libMesh. All simulations are run on a local Linux workstation with eight Intel(R) Xeon(R) CPU cores (2.65 GHz) and 32 GB RAM, the simulation time for one LV model with one set of passive and active parameters is around 24 hours.

S0.2 Optimization results

Results on optimization and strain comparisons between measurements and model predictions using optimized parameters are summarized in Table S1, including end-diastolic (ED) volume, end-systolic (ES) volume and systolic strain. The relative difference in volume is defined as

$$\Delta\% = \frac{\sum_i^N (|V_i - V_i^{\text{measured}}|/V_i^{\text{measured}})}{N} \times 100, \tag{S9}$$

in which i is the subject number, N is the total number of subjects either in healthy volunteers (HV) or in the MI group, V_i is ED volume or ES volume.

Similarly for systolic strain difference

$$\Delta\% = \frac{\sum_i^N (|\varepsilon_i - \varepsilon_i^{\text{measured}}|/|\varepsilon_i^{\text{measured}}|)}{N} \times 100, \quad \text{(S10)}$$

in which ε_i is the average circumferential strain in systole related to end-diastole in i^{th} subject, ε_i is defined as $\frac{\sum_l^L \varepsilon_i^l}{L}$, and L is the total number of segments for measuring strain.

Table S1: Summary of optimization result

	ED volume (mL)			ES volume (mL)			systolic strain (%)		
	simulated	measured	$\Delta\%$	simulated	measured	$\Delta\%$	simulated	measured	$\Delta\%$
HV	126±21	127±21	2±2%	56±14	55±14	2.3±2%	-0.19±0.02	-0.18±0.02	8±5%
MI	144±27	145±26	1.4±1%				-0.16±0.01	-0.16±0.01	1.1±1%

HV: healthy volunteers, MI: myocardial infarction patients. ES volume is not compared in MI patients because of entirely non-contractile assumption in the MI region.

S0.3 Biomechanical features and correlation analysis

Our feature selection has two phases: a PRE-SELECTION, based on physiological criteria and linear correlation analysis, and a POST-SELECTION, based on multivariate statistics and machine learning. In specific, the pre-selection is performed based on linear correlation analysis among biomechanical factors (T_a, T_{req}, $T_{\text{a}}^{\text{norm}}$, C^{s} and σ_{f}), and their associations to CMR measurements (EDV: end-diastolic volume, LVEF: left ventricular ejection fraction, CS: systolic circumferential strain, ESV: end-systolic volume, and SBP: systolic blood pressure), as shown in Table S2. If two features have very strong correlation both in the MI group and the healthy group, and both correlate to other features in a similar way, then only one feature will be selected. The correlation analysis shows that σ_{f} and T_a are highly correlated. T_a is selected in this study because σ_{f} is less reliable due to the assumption of end-diastolic pressure. Although C^{s} is correlated to T_a in both groups, they relate to CMR measurements in different ways, therefore, C^{s} is included. Similarly, for T_{req} and $T_{\text{a}}^{\text{norm}}$. For the post-selection based on multivariate analysis, the fact that some features are not independent is not a problem, as this can be dealt with by the automatic relevance determination (ARD) method of the Gaussian process, refer to Section 6.4 of C.M. Bishop's book [4] for details.

Table S2: Linear correlation analysis among biomechanical features and their correlations to CMR measurements

Healthy Volunteers

	T_a	T_{req}	T_a^{norm}	C_s	σ_f	EDV	LVEF	CS	ESV	SBP
T_a	1	0.9 (<0.01)	0.37 (0.06)	0.64 (<0.01)	0.99 (<0.01)	0.22 (0.03)	-0.09 (0.64)	-0.04 (0.86)	0.23 (0.24)	0.77 (<0.01)
T_{req}	0.9 (<0.01)	1	0.14 (0.49)	0.25 (0.22)	0.85 (<0.01)	0.10 (0.61)	0.09 (0.64)	-0.17 (0.4)	0.13 (0.53)	0.82 (<0.01)
T_a^{norm}	0.37 (0.06)	0.14 (0.49)	1	0.55 (0.002)	0.46 (0.02)	0.59 (0.001)	-0.66 (<0.01)	0.51 (<0.01)	0.63 (<0.01)	-0.30 (0.13)
C_s	0.64 (<0.01)	0.25 (0.22)	0.55 (0.002)	1	0.71 (<0.01)	0.30 (0.13)	-0.40 (0.04)	0.23 (0.25)	0.30 (0.14)	0.28 (0.16)
σ_f	0.99 (<0.01)	0.85 (<0.01)	0.46 (0.02)	0.71 (<0.01)	1	0.29 (0.14)	-0.19 (0.34)	0.05 (0.79)	0.31 (0.12)	0.70 (<0.001)

MI Patients

	T_a	T_{req}	T_a^{norm}	C_s	σ_f	EDV	LVEF	CS	ESV	SBP
T_a	1	-0.30 (0.37)	0.59 (0.054)	0.79 (<0.01)	0.96 (<0.01)	0.23 (0.5)	-0.39 (0.24)	0.26 (0.44)	0.23 (0.51)	0.68 (0.02)
T_{req}	-0.30 (0.37)	1	-0.30 (0.38)	-0.80 (<0.01)	-0.40 (0.22)	-0.28 (0.41)	0.44 (0.18)	-0.72 (0.01)	-0.25 (0.45)	-0.08 (0.82)
T_a^{norm}	0.59 (0.054)	-0.30 (0.38)	1	0.57 (0.07)	0.53 (0.09)	0.47 (0.15)	-0.59 (0.06)	0.28 (0.41)	0.48 (0.13)	-0.18 (0.6)
C_s	0.79 (<0.01)	-0.80 (<0.01)	0.57 (0.07)	1	0.83 (<0.01)	0.40 (0.22)	-0.48 (0.13)	0.63 (0.04)	0.39 (0.24)	0.43 (0.19)
σ_f	0.96 (<0.01)	-0.40 (0.22)	0.53 (0.09)	0.83 (<0.01)	1	0.30 (0.37)	-0.43 (0.18)	0.28 (0.40)	0.29 (0.38)	0.70 (0.02)

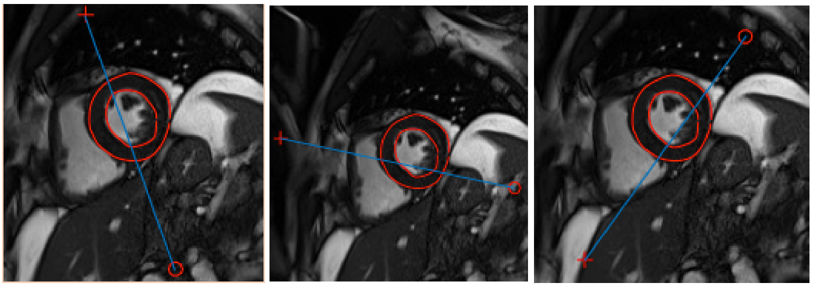

(a)

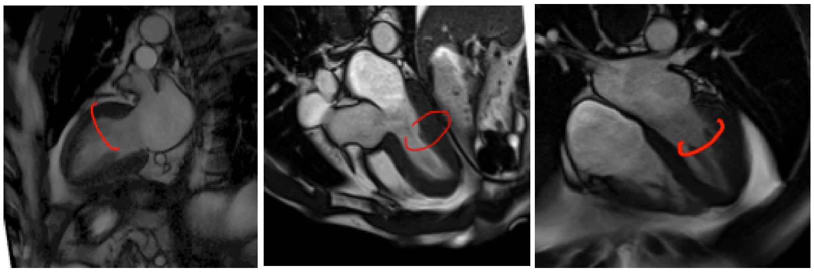

(b)

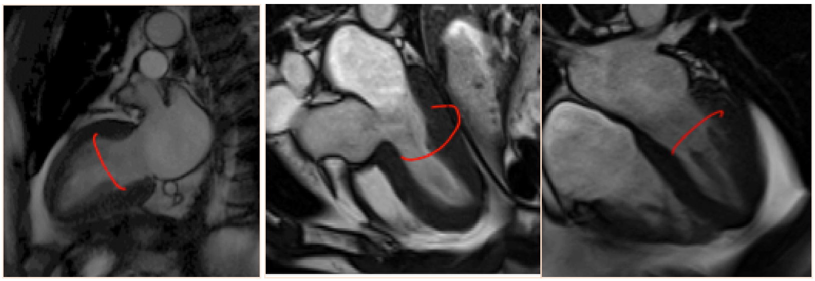

(c)

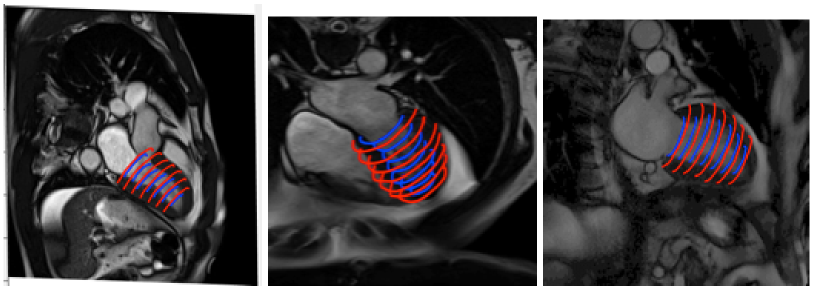

(d)

Fig. S1: Realignment of ventricular boundaries from short-axis veiws. (a) Segmentation of ventricular boundaries in short-axis views, from left to right: one-chamber view, LVOT view and four-chamber view. The blue lines represents the intersction line between the short-axis views and the correponding long-axis views. (b) Superimposing ventricular boundaries from short-axis views into long-axis views, only endocardial boundary is showed; (c) motion correction by translating short-axis boundaries along the intersection line according to the three long-axis views; (d) LV boundaries after motion correction superimposed in long-axis views.

S0.4 Full Feature Selection Results

Here, we present the full feature selection results, per data set and per method, from which Figure 8 in the main paper was obtained. The importance measure of Lasso is expressed as the absolute value of the average regression coefficients shown in the top panel of Fig. S2. Dataset D_1 in the left column of Fig. S2 indicates that the factors C^{s}, $T_{\mathrm{a}}^{\mathrm{norm}}$, and T_a have the importance corresponding to high average coefficients, whereas EDV and SBP have the lowest relevance. The plot for dataset D_2 (middle column) shows T_a and SBP with the highest importance. The two ratios C^{s} and $T_{\mathrm{a}}^{\mathrm{norm}}$ are missing from this dataset. For D_3 only the factor $T_{\mathrm{a}}^{\mathrm{norm}}$ exhibits a clear relevance. The previously relevant C^{s} (in dataset D_1) has little importance, suggesting an interaction effect with T_{req}, which is missing from dataset D_3.

For GP-ARD, the importance measures are expressed as the inverted and normalised length scales, shown in the second row of Fig. S2. For dataset D_1, the most relevant factors are T_{req}, C^{s}, and $T_{\mathrm{a}}^{\mathrm{norm}}$. This finding is coherent with Lasso, except that T_{req} is more relevant and not T_a. T_{req} has the highest relevance for dataset D_2 and SBP and T_a are second. In the third dataset D_3, the ratios C^{s} and $T_{\mathrm{a}}^{\mathrm{norm}}$ have the highest importance together with T_a. In contrast to Lasso, the importance of C^{s} does not decrease, although T_{req} is missing from this dataset. To illustrate the relationship of the three most important factors to their outcome, we plotted posterior probabilities as a contour plot with corresponding class labels for dataset D_1. Fig. 11 shows the most relevant factor C^{s} plotted against T_{req} and against $T_{\mathrm{a}}^{\mathrm{norm}}$. The first plot shows a high non-linearity in the relation between the two factors and their respective outcomes. The decision boundary at 0.5 separates the two classes in a narrow band. There is a high risk of misclassification for factor values that are in the close vicinity of this boundary. Nonetheless, GP-ARD captures the non-linearity of these class distributions sufficiently good. One exception is shown in the right panel of Fig. 11 with the circle located in the middle of the cloud of crosses, which might represent a high error measurement.

The Decision Tree provides the usage metric to quantify factor importance as shown in the third row of Fig. S2. For both datasets, D_1 and D_3, only EDV with 20% and $T_{\mathrm{a}}^{\mathrm{norm}}$ with 100% are used for tree construction. It is surprising that EDV is relevant in these cases because EDV is not indicated as an important factor by the other methods, except for the Random Forest in dataset D_3. In addition, C^{s} is not used at all in the tree, which is also surprising given that it is relevant for Lasso and GP-ARD, and to some extent for the Random Forest (see below). For dataset D_2 all factors have equal importance.

The Random Forest provides the mean decrease of accuracy as importance measure, shown in the bottom row of Fig. S2. The variable $T_{\mathrm{a}}^{\mathrm{norm}}$ has the highest importance in the datasets in which it is present (D_1 and D_3). C^{s} is less important compared to Lasso and GP-ARD, but the relevance of SBP is more expressed compared to the remaining methods.

S0.5 Description of Classification Methods

The following section describes each of the machine learning and statistical methods that we use for the prediction of myocardial contractile function and for the identification of relevant features. See Section 2.3 for details on method application and evaluation.

Notation We will use n for the number of observations in the data set, and m as the number of features that we use as predictor variables. The response vector with the class labels is denoted with $\boldsymbol{y} = (y_1, y_2, \ldots, y_n)^\intercal$. A response is coded with the binary 0/1, such that a variable $Y = 0$ defines a healthy volunteer, and $Y = 1$ defines a MI patient. The symbol $\boldsymbol{X}$ is an $n \times m$ matrix that contains the predictor variables x_{ij} with the samples in the rows and the features in the columns. The vector $x_i = (x_{i1}, x_{i2}, \ldots, x_{im})$ refers to the variable measurements of sample $i \in \{1, \ldots, n\}$ and contains m features. The vector $\boldsymbol{x_j} = (x_{1j}, x_{2j}, \ldots, x_{nj})^\intercal$ holds all samples of the feature $j \in \{1, \ldots, m\}$. Whenever a method uses logistic regression we denote the coefficients with $\boldsymbol{\beta} = (\beta_0, \beta_1, \beta_1, \ldots, \beta_m)^\intercal$. In this case, we expand the design matrix $\boldsymbol{X}$ with an initial column $\boldsymbol{x}_0 = (1, \ldots, 1)$ that corresponds to the intercept. Note that all vectors are in bold face except the feature vector x_i.

Logistic Regression with GLMs Generalized Linear Models (GLM) extend ordinary linear regression with support for arbitrary distributions for the response, rather than assuming a simple normal distribution. In addition, the linear predictor $\boldsymbol{X\beta}$ is used to determine the mean μ of the distribution through a (vector) link function $\mu = g^{-1}(\boldsymbol{X\beta})$, where the coefficient $\boldsymbol{\beta}$ is unknown. The link function provides a non-linear mapping of the predictors to the expected value of the dependent variables. GLMs support logistic regression by specifying a binomial distribution and a logit link function defined as $\ln(\mu/1-\mu)$. The mean vector μ is defined as $\mu = (\boldsymbol{1} + \exp(-\boldsymbol{X\beta}))^{-1}$. The unknown coefficients, $\boldsymbol{\beta}$, are typically estimated with maximum likelihood. We use the R function `glm()` with the family `binomial` that implies a logit link function. For the univariate logistic regression, we apply each feature j as a single predictor variable to establish a benchmark for the remaining methods that use multiple predictors. Also, we evaluate multivariate logistic regression that includes all features as predictor variables.

Linear Discriminant Analysis The method of linear discriminant analysis (LDA) attempts to seperate two or more classes by the linear combination of features that best characterize each class. For two classes $y_i = 0$ and $y_i = 1$, and a set of features denoted by x_i, LDA assumes that the conditional probabilities $p(x_i|y_i = 0)$ and $p(x_i|y_i = 1)$ are both normally distributed with mean and covariance parameters $(\boldsymbol{\mu}_{y=0}, \Sigma)$ and $(\boldsymbol{\mu}_{y=1}, \Sigma)$ [5]. The covariance matrix is assumed to be identical for both classes with full rank. To determine if a feature vector x_i is a good predictor for response $y_i = 0$ or $y_i = 1$, x_i is projected onto

a vector $\omega = \Sigma(\boldsymbol{\mu}_{\mathbf{y=1}} - \boldsymbol{\mu}_{\mathbf{y=0}})$. This vector determines the direction on the hyperplane c that separates the two classes $y = 0$ and $y = 1$. The hyperplane c is defined by a certain threshold T and the mean and covariance of the classes with

$$c = 1/2(T - \boldsymbol{\mu}_{\boldsymbol{y=0}}^{\intercal}\Sigma^{-1}\boldsymbol{\mu}_{\boldsymbol{y=0}} + \boldsymbol{\mu}_{\boldsymbol{y=1}}^{\intercal}\Sigma^{-1}\boldsymbol{\mu}_{\boldsymbol{y=1}}) \tag{S11}$$

The class assignment for x_i is thus a decision criteria with $x_i \cdot \omega < c$. We use the R package *MASS* with the function `lda()` to assess the performance of LDA.

Sparse Logistic Regression with Lasso The Lasso (least absolute shrinkage and selection operator) is a sparse regression method that was initially applied to ordinary least squares (OLS) regression [6]. The method shrinks the regression coefficients $\boldsymbol{\beta}$ of the m features towards zero using a regularization term that penalizes high coefficient values. Lasso can be applied to class prediction by transformation into ordinary logistic regression in conjunction with a L1-norm penalty [7]. This can be expressed as a penalized log-likelihood $L(\boldsymbol{\beta})$ that has an additional regularization term dependent on the penalty parameter λ:

$$L(\boldsymbol{\beta}) = -\sum_{i=1}^{n}(1 - y_i)\boldsymbol{\beta}^{\intercal}\boldsymbol{x}_i + \ln(1 + \exp(-\boldsymbol{\beta}^{\intercal}\boldsymbol{x}_i)) - \lambda\sum_{j=1}^{m}|\beta_j| \tag{S12}$$

The last term sums the absolute coefficient values weighted by λ, which can be selected with cross-validation or an information criterion. By maximizing $L(\boldsymbol{\beta})$, the vector of coefficients $\boldsymbol{\beta}$ is simultaneously shrunk and pruned, which has an effect similar to feature selection. We use the R package *glmnet* with function `glmnet()` in the binomial family, and LOOCV to assess the methods performance. In each LOOCV iteration, we apply 5-fold cross-validation with the function `cv.glmnet()` to determine an appropriate λ before making a prediction using the optimized coefficients $\boldsymbol{\beta}$. The data is standardized in the internal procedure of `glmnet()`. We found that subsequent applications of LOOCV tend to produce different predictions, which is caused by significant deviations in the optimised λ penalty values, as can be seen in Fig. S3. To overcome this problem we apply bootstrapping with 20 repetitions for the prediction, and 100 repetitions for the coefficient estimates that are used as an importance measure. We also applied the Elastic Net method, which combines the L1 penalty term from Lasso with the L2 penalty term from ridge regression, but found little difference in prediction accuracy compared to Lasso [8].

K-Nearest Neighbours The K-Nearest Neighbour (KNN) method is a non-parametric classifier that does not rely on prior assumptions about the underlying variable distribution [9]. Instead, it uses a metric such as the Euclidian distance to determine the predominant class label of training observations located

in the neighbourhood of a test observation. The class that has the majority is assigned to the test observation. The size of the neighbourhood is defined by the k nearest neighbours of the training set, where $k \in \{1, .., n-1\}$, and n is the number of observations in the training set. The R package *class* with the function `knn()` was used to run the method. The data is z-score transformed since some of the variables are on different scales and KNN uses a distance measure to compare pairs of observations.

The setting of k can significantly influence prediction accuracy. To determine a $k \in \{1, \ldots, n-1\}$ with the highest prediction accuracy, we apply LOOCV to each setting of k and calculate the corresponding misclassification error rate, sensitivity, and specificity. Fig. S5 shows an overview of these measures given different k for the three datasets D_1, D_2, and D_3. The vertical lines highlight the k that minimises the error rate, which is $k = 1$ for all datasets. This small value of k can indicate a very narrow boundary between the two classes or a highly non-linear relationship between predictors and class labels.

Decision Trees A decision tree is a graph with a tree structure that represents a flow of subsequent tests on the input data with a final assignment of class labels [10]. The head node and each of the internal nodes of the tree is associated with rules on the feature $j \in \{1, \ldots, m\}$ that divide the data into smaller fragments. The leaf nodes of the tree represent the class labels y_i. Given a test observation, the tree is traversed from the head node down to the leaf nodes. A child node is determined by testing the feature value of the input against the rule of the parent node. Decision trees have the advantage that only essential features are considered, and they provide an intuitive understanding of how the data is structured if the tree is simple enough.

A well-established implementation of constructing a decision tree is the C5.0 algorithm [11]. C5.0 uses a gain of information criterion defined as $[E(S_1) - E(S_2)]$, which measures the entropy difference after splitting a node S_1 into the child nodes S_2.

$$E(S) = -\sum_{j=1}^{m} p_j \log(p_j) \tag{S13}$$

where p_j is the probability of assignment of a case to leaf node j.

The split at a value of feature i is more likely if the difference of entropy E in the partition S_1 before the split is large compared to the entropy of the partitions S_2 after the split. Since S_2 has multiple partitions $P_{\{1,\ldots,p\}}$, the entropies of each partition of S_2 are weighted with a parameter ω: $E(S_2) = \sum_{l=1}^{p} \omega_l E(P_l)$. The risk of overfitting is reduced with back pruning the fully constructed tree in dependence of a cost penalty parameter. We use the R package *C50* with varying penalty and boosting setups to assess the method. For the final evaluation we apply nested LOOCV that estimates the best parameter setup for each single prediction, such that a prediction sample is never part of a training set. Note that the decision tree implementation with the *C50* R package provides two

metrics that quantify the importance of the factors with the function `C5imp()`. The default 'usage' metric of a factor determines the percentage of observations that are assigned to a class label based on a split of the specific factor. We use this metric as the indicator of factor importance in predicting the outcome. Two importance measures were determined with the same parameter setup used for classification. Note that the usage metrics from the individual trees of the boosting trials are merged into the final usage statistic.

The Decision Tree C5.0 algorithm uses back pruning of the fully constructed tree to decrease the chance of overfitting that can be caused by very detailed and local decision rules. The cost penalty parameter controls the amount of pruning. Also, the C5.0 algorithm supports boosting, which uses several trees for prediction. First, we evaluate the misclassification error rate given different settings for the penalty cost and the amount of boosting. Fig. S7 shows the error rate for increasing penalty cost with four different boosting setups: no boosting (1), 10, 20, and 30 trials as indicated in the legend. The vertical line highlights the cost penalty with the lowest error. The plots in Fig.9 show the corresponding decision trees. Note that only the first tree from the boosting set is displayed for each dataset. The first and third tree are equivalent because the same subset of features is selected for construction of the first tree. Furthermore, Fig. S6 in the Appendix shows the ROC plots for each of the C5.0 trees that differ in the penalty and boosting setup. The distribution of ROC points indicates that there is a substantial difference between a lack of boosting (upper panels) and the remaining setups. Although the difference in AUROC is marginal for the data set D_2, there is a steep increase of classification performance for D_1 using 30 boosting trials instead of a lower number.

However, these observations are overoptimistic because each test sample was also used for training in the LOOCV. For the final performance measure we instead apply a nested LOOCV approach: In each prediction of a class label i, we estimate the best boosting and penalty parameter given the $(n-1)$ remaining training samples with an inner LOOCV, such that we have $n-2$ training samples. Thus the C5.0 parameters can change for every prediction, and the prediction samples are never part of a training set.

Random Forests A Random Forest classifier consists of an ensemble of decision trees that is constructed with bagging in combination with random feature sub-selection, i.e. feature bagging. The original method used only ordinary bagging to build trees based on random samples from the training set [12], and averaging the class predictions from the set. However, the single application of bagging will tend to produce highly correlated trees whenever there is a dominance of a few essential features. The addition of feature sub-selection to the algorithm can reduce correlation and is applied to every split of the node: At each split a subset of the available features is selected. One of these features and a specific value is chosen to generate the child nodes [13]. Multiple trees constructed in such a way are more heterogenous and uncorrelated compared to trees using the

full feature set. The average or majority vote of all trees is used as the final class prediction. We use the R package *randomForest* with 100 bagging trees.

For the importance measure of each factor, we use the mean decrease of accuracy that is provided with the package *randomForest.* This metric calculates the difference in error rate between the standard forest and a forest with randomly permuted factor observations. The error rate increases when permuting a feature that is relevant, and thus the accuracy of prediction decreases. In contrast, if the error rate drops for a particular permuted factor, there is an indication of an adverse effect on class prediction.

The Random Forest package *randomForest* provides a choice for the number of trees in the forest and the number of features that are sub-selected in each split. However, there is no point in attempting to estimate the best parameters based on the error rate because the features are selected randomly. Since the prediction depends on this selection, the accuracy will also vary under the same parameters. To illustrate this, Fig. S8 shows the error rates for varying forest sizes and feature sub-selections. The error lines are considerably flat and the best parameter setting highlighted with a vertical line will change in every execution. For the classification assessment, we use the default setting of *randomForest*, which is $\sqrt{p}$ for the number of features in each split, and a size of 500 trees. The output is either an aggregated class probability or an individual prediction of each tree. We applied a majority vote over the particular tree predictions and found no difference in the aggregated probabilities under a decision boundary of 0.5.

Gaussian Process with Automatic Relevance Determination A detailed treatment of Gaussian processes (GPs) can be found in [14], and we only summarize the essential ideas. A GP defines a prior distribution over functions, which is converted into a posterior distribution once data have been observed. Formally, a GP is a stochastic process such that the join distribution of any finite subset is multivariate Gaussian. The joint Gaussian prior $p(f|\boldsymbol{y}, \boldsymbol{x}) = p(f(x_1), \ldots, f(x_m)|\boldsymbol{y}, \boldsymbol{x})$ is fully defined by a mean, which is often assumed to be zero, and a covariance matrix K, which is calculated with a kernel function $k(x_p, x_q)$ such that $f \sim \mathcal{N}(\mathbf{0}, K)$. The elements $p, q \in \{1, \ldots, m\}$ of the covariance matrix K are typically chosen in such a way that the outputs (y_p, y_q) are stronger correlated for similar input points x_p and x_q. The squared exponential (SE) kernel is a popular choice for the covariance. The function has a variance parameter σ and a length-scale parameter l that controls the similarity of data points. By including individual length-scale parameters $(l_1, \ldots, l_m)$ for each of the input variables $(x_1, \ldots, x_m)$, the SE kernel can be used for automatic relevance determination:

$$k(x_p, x_q) = \sigma^2 \exp\left(-\sum_{j=1}^{m} \frac{(x_{pj} - x_{qj})^2}{2l_j} \right) \tag{S14}$$

Thus, a low length scale will result in a large influence of the associated variable, and a high length scale means a low relevance for predicting the outcome.

A GP can be used for classification with the logistic link function $\lambda(\boldsymbol{x}^{\intercal}\beta) = 1/(1+\exp(-\boldsymbol{x}^{\intercal}\beta))$. The un-normalized GP posterior composed of logistic likelihood and GP prior is analytically intractable, but the log posterior is concave. Hence, approximated inference is feasible with a standard procedure such as the expectation propagation algorithm (EP). Another option is the probit function for the likelihood, which is very similar to the logit distribution, but with minor difference in the tail. We evaluated both likelihoods together with EP, Laplace approximation and a Variational Bayesian method for the inference of the hyper-parameters. Based on the error rates shown in Fig. S4 we selected logit with EP for all further studies. We use the Matlab package *gpml* [15] to train a GP and make predictions using the likelihood `likLogistic()`, covariance `covSEard()`, and the inference function `infEP()`. Note that we also use the length scales l_j as indicators for the importance of each factor $j = (1, \ldots, m)$. They were normalized with the variable range and inverted with $(1-(l_j/(max(x_j)-min(x_j))))$. This transformation renders the length parameter coherent to the importance measures of the other methods such that high values indicate high importance or relevance.

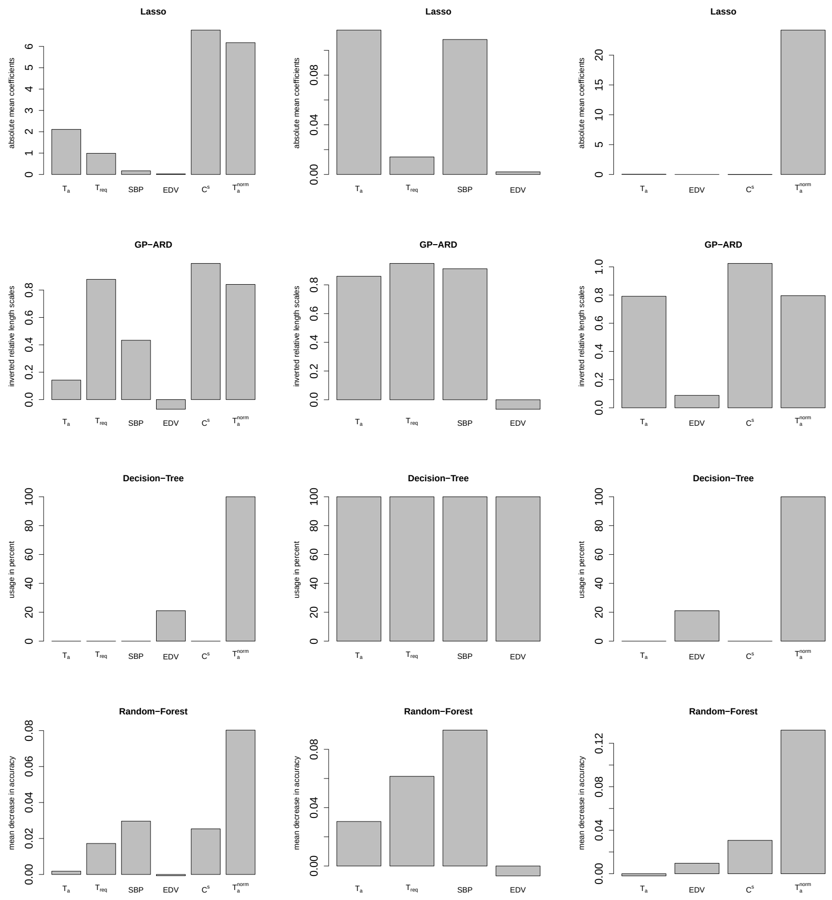

Fig. S2: Importance of features for individual methods: GP-ARD, Lasso, Decision Tree and Random Forest. Each column corresponds to the three different datasets D_1 (left), D_2 (centre), and D_3 (right). Taller bars indicate more important features. A summary plot that shows the ranked contributions of each method is displayed in Fig. 8.

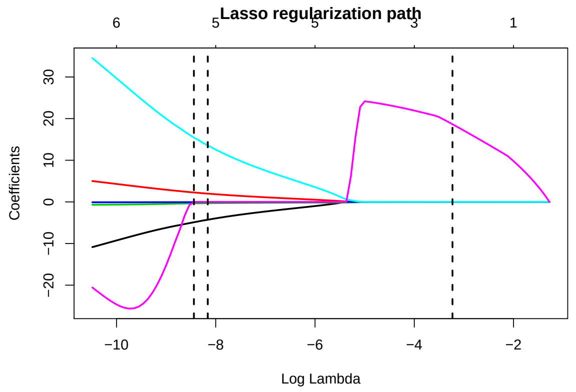

Fig. S3: Lasso regularisation path of the regression coefficients with different optimised penalty parameters (vertical lines) for dataset D_1. Each line corresponds to one coefficient and one feature. The different penalties cause substantial deviations in the selected coefficient values. The reason for this is the randomness of the training set selection of the Lasso cross-validation procedure. However, averaging over several bootstrap samples of the Lasso yields a more robust estimate of the coefficients.

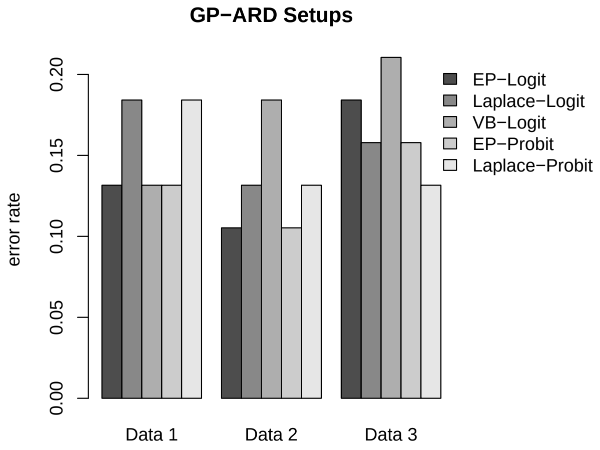

Fig. S4: GP-ARD with different setups for the likelihood (logit and probit), and the inference method that optimises the hyperparameters: EP (Expectation-Propagation), Laplace, and VB (Variational Bayesian). The error rates are derived from LOOCV for each of the three datasets D_1, D_2, and D_3.

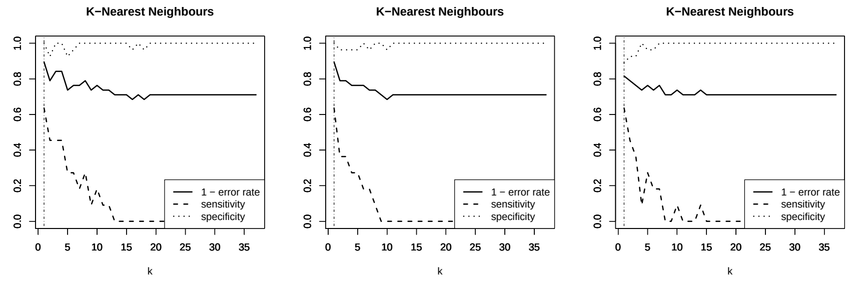

Fig. S5: K-nearest neighbours (KNN) with varying parameter $k \in (1, \ldots, n-1)$ and corresponding change in error rate, sensitivity, and specificity. The scores for each k were determined with out of sample cross-validation using LOOCV. The left, middle and right columns correspond to the datasets D_1, D_2 and D_3, respectively.

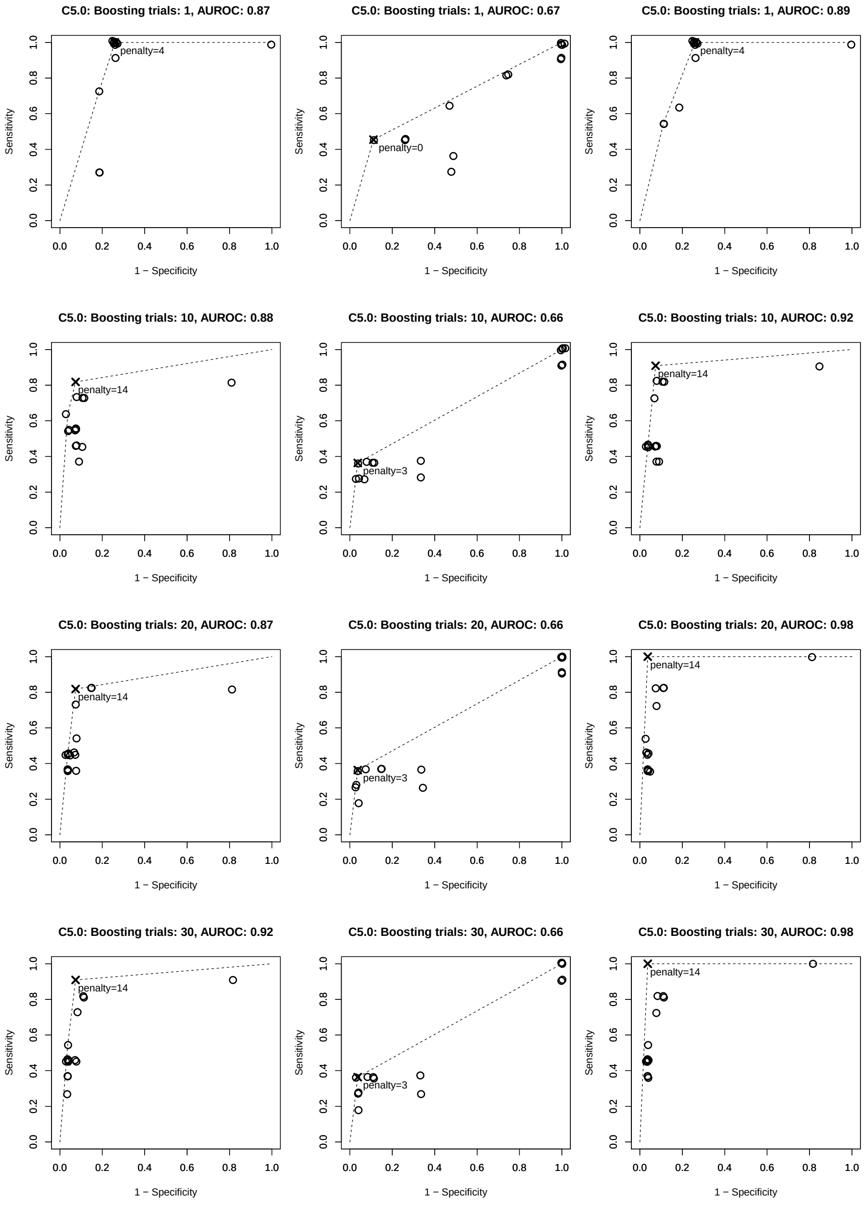

Fig. S6: **C5.0 Decision Tree configurations:** Different settings for boosting trials and the cost penalty for three different datasetups displayed in each column. The upper row shows ROC plots that lack boosting, and the lower row uses boosting with 10 trials. Each symbol in the ROC plots corresponds to a specific cost penalty. The left top most point is marked with a cross. LOOCV was used for each setting of boosting and penalty to estimate the sensitivity and specificity measures.

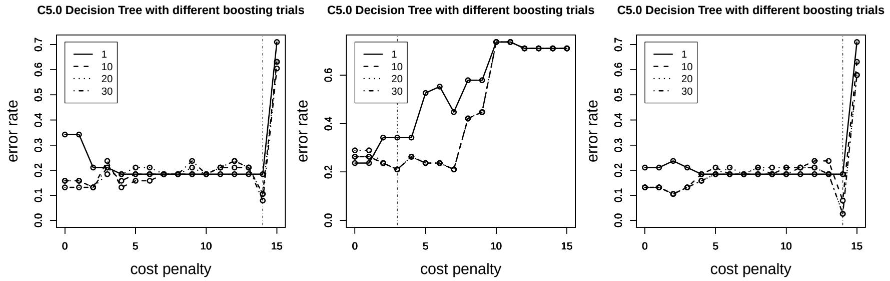

Fig. S7: Misclassification error rate for the C5.0 Decision Trees for three different datasets (columns), each with varying error penalty (x-axis) and boosting trials (line style). These plots correspond to the ROC plots in Fig. S6 but with an error rate as the indicator of prediction accuracy. The vertical lines indicate the lowest error. The corresponding cost penalty and number of boost trials is selected for the final evaluation.

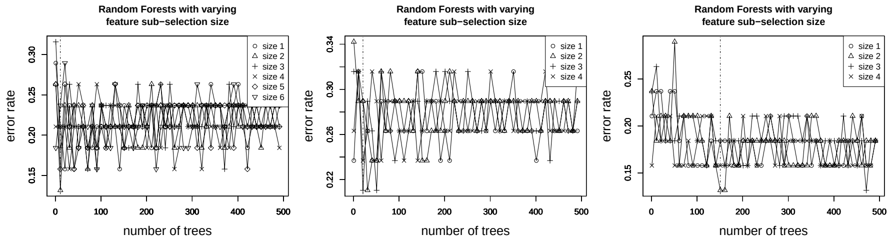

Fig. S8: Error rates of Random Forest given varying forest and feature sub-selection sizes. Each panel corresponds to one of the three datasets. The vertical lines indicate the setup with the lowest error rate. However, in each execution, these plots and the best setup will change due to the randomness of the feature selection. Thus we use the default settings provided with the R package *randomForest*.

References

1. Carrick, D., Oldroyd, K., McEntegart, M., Haig, C., Petrie, M., Eteiba, H., Hood, S., Owens, C., Watkins, S., Layland, J., et al.: A randomized trial of deferred stenting versus immediate stenting to prevent no-or slow-reflow in acute st-segment elevation myocardial infarction (defer-stemi). Journal of the American College of Cardiology **63**(20) (2014) 2088–2098
2. Peskin, C.: The immersed boundary method. Acta numerica **11** (2002) 479–517
3. Griffith, B.E., Luo, X.Y.: Hybrid finite difference/finite element version of the immersed boundary method. eprint from arXiv (url: https://arxiv.org/abs/1612.05916) (2016)
4. Bishop, C.: Pattern Recognition and Machine Learning. Springer-Verlag New York (2006)
5. Venables, W., Ripley, B.: Modern applied statistics with S-PLUS. Springer Science & Business Media (2013)
6. Tibshirani, R.: Regression shrinkage and selection via the lasso. Journal of the Royal Statistical Society. Series B (Methodological) (1996) 267–288
7. Lee, S.I., Lee, H., Abbeel, P., Ng, A.: Efficient l˜ 1 regularized logistic regression. In: Proceedings of the National Conference on Artificial Intelligence. Volume 21., Menlo Park, CA; Cambridge, MA; London; AAAI Press; MIT Press; 1999 (2006) 401
8. Sampson, R.: Identifying Left-Ventricular Myocardial Infarction: a preliminary investigation of new biomechanical variables in a case-control study. MSci dissertation, University of Glasgow (2016)
9. Altman, N.S.: An introduction to kernel and nearest-neighbor nonparametric regression. The American Statistician **46**(3) (1992) 175–185
10. Quinlan, J.R.: Simplifying decision trees. International journal of man-machine studies **27**(3) (1987) 221–234
11. Wu, X., Kumar, V., Quinlan, R., Ghosh, J., Yang, Q., Motoda, H., McLachlan, G., Ng, A., Liu, B., Philip, Y., et al.: Top 10 algorithms in data mining. Knowledge and information systems **14**(1) (2008) 1–37
12. Ho, T.K.: Random decision forests. In: Document Analysis and Recognition, 1995., Proceedings of the Third International Conference on. Volume 1., IEEE (1995) 278–282
13. Friedman, J., Hastie, T., Tibshirani, R.: The elements of statistical learning. Volume 1. Springer series in statistics Springer, Berlin (2001)
14. Rasmussen, C.E., Williams, C.K.I.: Gaussian Processes for Machine Learning. MIT Press (2006)
15. Rasmussen, C.E., Nickisch, H.: Gaussian processes for machine learning (gpml) toolbox. Journal of Machine Learning Research **11**(Nov) (2010) 3011–3015
